# Usefulness of microsatellite loci for differentiating between *Dibothriocephalus dendriticus* and *Dibothriocephalus ditremus* (Cestoda: Diphyllobothriidea)

**DOI:** 10.1051/parasite/2025033

**Published:** 2025-07-04

**Authors:** Ivica Králová-Hromadová, Lucia Dinisová, Alžbeta Radačovská, Egil Karlsbakk, Karl Skírnisson, Eva Čisovská Bazsalovicsová

**Affiliations:** 1 Institute of Parasitology, Slovak Academy of Sciences Hlinkova 3 04001 Košice Slovakia; 2 The University of Veterinary Medicine and Pharmacy in Košice Košice Slovakia; 3 Department of Biological Sciences, University of Bergen P.O. Box 7803 N-5020 Bergen Norway; 4 Institute for Experimental Pathology, University of Iceland Keldnavegur 3 IS 112 Reykjavík Iceland

**Keywords:** Fish tapeworms, Molecular taxonomy, Microsatellite loci, Species-specific primers

## Abstract

Differentiating between two diphyllobothriid tapeworms *Dibothriocephalus dendriticus* and *Dibothriocephalus ditremus* is complicated due to their morphological plasticity, intraspecific variability and a wide range of common hosts. The aim of this study was to develop a species-specific PCR-based method for single-step discrimination between *D. dendriticus* and *D. ditremus*. Intraspecific variation and interspecific differences were analysed in subunits/spacers of nuclear rRNA genes and protein-coding genes of mitochondrial DNA. In addition, the specificity of primers designed for the amplification of microsatellite loci in *D. dendriticus* was tested on *D. ditremus* DNA. Due to high identity within the rRNA gene in these species, no suitable DNA regions could be identified for the design of the species-specific primers. A higher level of interspecific differences was detected in the mitochondrial *cox*1 and *cob* genes, in which regions containing species-specific mutations were chosen for the design of *D. dendriticus*- and *D. ditremus*-specific primers. However, their specificity was not confirmed, as the *D. dendriticus*-specific primers also annealed to *D. ditremus* DNA and *vice versa*. Of the 15 primer pairs designed for the amplification of microsatellite loci in *D. dendriticus,* 13 primer pairs also annealed to *D. ditremus* DNA. Only two primer pairs, which amplify the Dd_8 and Dd_33 loci have been proven to be *D. dendriticus*-specific. The effectiveness and high reproducibility of the Dd_8 primers were validated on ~3,500 *D. dendriticus* and *D. ditremus* plerocercoids from Iceland and Norway. These primers are recommended for future molecular differentiation between both *Dibothriocephalus* species.

## Abstract (fr)

La différenciation entre deux cestodes Diphyllobothriidae, *Dibothriocephalus dendriticus* et *Dibothriocephalus ditremus*, est complexe en raison de leur plasticité morphologique, de leur variabilité intraspécifique et de la grande diversité de leurs hôtes communs. L’objectif de cette étude était de développer une méthode de PCR spécifique à chaque espèce pour la discrimination en une seule étape entre *D. dendriticus* et *D. ditremus*. La variation intraspécifique et les différences interspécifiques ont été analysées dans les sous-unités/espaceurs des gènes de l’ARNr nucléaire et dans les gènes codant pour des protéines de l’ADN mitochondrial. De plus, la spécificité des amorces conçues pour l’amplification des loci microsatellites chez *D. dendriticus* a été testée sur l’ADN de *D. ditremus*. En raison de la forte identité au sein du gène d’ARNr chez ces espèces, aucune région d’ADN appropriée n’a pu être identifiée pour la conception d’amorces spécifiques à l’espèce. Un niveau plus élevé de différences interspécifiques a été détecté dans les gènes mitochondriaux *cox1* et *cob*, dans lesquels des régions contenant des mutations spécifiques à l’espèce ont été choisies pour la conception d’amorces spécifiques à *D. dendriticus* et *D. ditremus*. Cependant, leur spécificité n’a pas été confirmée, car les amorces spécifiques à *D. dendriticus* s’hybrident également à l’ADN de *D. ditremus* et *vice versa*. Sur les 15 paires d’amorces conçues pour l’amplification des loci microsatellites chez *D. dendriticus*, 13 paires d’amorces s’hybrident également à l’ADN de *D. ditremus*. Seules deux paires d’amorces, qui amplifient les loci Dd_8 et Dd_33, se sont avérées spécifiques à *D. dendriticus*. L’efficacité et la reproductibilité élevée des amorces Dd_8 ont été validées sur ~3 500 plérocercoïdes de *D. dendriticus* et de *D. ditremus* d’Islande et de Norvège. Ces amorces sont recommandées pour la différenciation moléculaire future des deux espèces de *Dibothriocephalus*.

## Introduction

The tapeworms *Dibothriocephalus dendriticus* (Nitzsch, 1824) (syn. *Diphyllobothrium dendriticum*) and *Dibothriocephalus ditremus* (Creplin, 1825) (syn. *Diphyllobothrium ditremum*) (Cestoda: Diphyllobothriidea) have sympatric distribution in the Arctic and subarctic regions of the Northern Hemisphere and an overlapping spectrum of intermediate and definitive hosts [5, 13, 22, 31]. *Dibothriocephalus dendriticus* has also been introduced into Patagonia in the southern cone region of South America, particularly in Chile [50] and Argentina [54]. Although the studied species are not closely related, they belong together with *Dibothriocephalus latus*, *Dibothriocephalus nihonkaiensis* and *Dibothriocephalus ursi* to the monophyletic genus *Dibothriocephalus*, which forms the most derived lineage within the family Diphyllobothriidae [64].

The life cycle of both species requires copepods and freshwater or anadromous fish as the first and the second intermediate hosts, respectively [36, 63]. The plerocercoids (the second larval stages) of both species are usually encapsulated in irregularly shaped capsules on the alimentary canal or the internal organs (stomach, liver, gonads, swim bladder, and peritoneum); rarely they can be found free in the heart or the body cavity of infected fish [5, 21, 22, 48]. The dominant intermediate hosts of both tapeworms are salmonids (family Salmonidae), mainly the genera *Coregonus* [7, 60], *Salvelinus* [23, 51], *Salmo* [62, 68], and *Oncorhynchus* [5, 42]. Plerocercoids were also found in fishes of the families Gasterosteidae, Cottidae, Osmeridae, and Lotidae [7, 42, 45, 59, 61]. The dominant definitive hosts of both tapeworms are piscivorous birds, mainly from the family Laridae [26, 55]. In addition, *D. dendriticus* can infect mammals, such as dogs [57], foxes [56], and bears [15], and is also one of the causative agents of dibothriocephalosis, a fish-borne parasitic zoonosis [33, 55].

The exact taxonomic identification of plerocercoids of both *Dibothriocephalus* species has been complicated due to the frequent co-infection in fish hosts [24, 37]. Both species can be histologically distinguished utilizing different microscopy techniques [5, 6, 21]. In other types of studies (*e.g.* ecology, distribution, genetics, *etc.*), plerocercoids have to be identified based on their external morphology using stereomicroscopy immediately after dissection of the fish.

The external morphology of the larvae of both *Dibothriocephalus* species, mainly their size and shape, may vary depending on methods of fixation, but also on the fish host, with the most profound differences between the infection of the three-spined stickleback (*Gasterosteus aculeatus*) and the brown trout (*Salmo trutta*) [5, 21]. As a result, the identification of larvae to the species level was not performed and larvae were assigned as “*Diphyllobothrium* spp.” in several studies from Europe, such as those conducted in Iceland [19, 32, 41] and Great Britain [28, 49], as well as in North America, including Canada [8, 20], and the USA [11, 65]. In addition, identification of parasitic material deposited in institutional or museum collections faces even greater challenges, since specimens have been collected and processed by various researchers using different procedures, fixatives and media. Adult tapeworms are sometimes fragmented, missing the scolex, or are immature, making a reliable taxonomic identification of species more difficult. According to our experience, adult tapeworms obtained from free-living carnivores are often decomposed due to prolonged time between death of the host and necropsy. Although plerocercoids of *D. dendriticus* and *D. ditremus* could be morphologically identified immediately after dissection [3, 4, 6], the identification of larvae (or their fragments) preserved in ethanol can be challenging.

Molecular tools have played an important role in the taxonomic identification of diphyllobothriid tapeworms, *e.g.* [39, 67, 68]. Wicht *et al.* [66] developed a set of four species-specific primers within the mitochondrial cytochrome *c* oxidase subunit 1 gene (*cox*1 mtDNA) to distinguish the most common diphyllobothriid species infecting humans, namely *D. latus, D. dendriticus, D. nihonkaiensis*, and *Adenocephalus pacificus* by multiplex PCR. The authors designed the *D. dendriticus*-specific primers, which amplified a 308 bp part of the *cox*1 gene specifically in *D. dendriticus.* A recent population genetic study on *D. dendriticus* showed that *D. dendriticus*-specific primers also yielded a non-specific amplification product for *D. ditremus* DNA [31]. This made the identification of *D. dendriticus* and its differentiation from *D. ditremus* based solely on PCR amplification unreliable and each PCR product had to be sequenced. However, sequencing increases financial costs and reduces time-effectiveness, particularly in population genetic studies involving several hundred individuals.

The aim of this study was to develop species-specific PCR-based method for single-step discrimination between *D. dendriticus* and *D. ditremus*. For this purpose, intraspecific variation and interspecific differences were analysed in the most frequently investigated DNA regions, the subunits and spacers of the nuclear ribosomal RNA (rRNA) genes and the protein-coding genes of mitochondrial DNA (mtDNA), which have been applied in studies on taxonomy, phylogeny and population genetics of Diphyllobothriidea. However, previously published studies were mainly focused on diphyllobothriideans infecting humans and *D. ditremus* was either not included in the analyses [2, 18, 46, 68], or only a limited number and selected populations of *D. ditremus* were analysed [1, 9, 38, 40, 58, 64]. Therefore, we decided to summarise all molecular data on *D. dendriticus* and *D. ditremus* to provide comprehensive analyses of the structure and variation of rRNA and mitochondrial genes and to assess their potential as discriminative markers. DNA regions without intraspecific variation but with substantial interspecific differences were selected as target regions for the design of primer sets, the specificity of which was validated on the DNA of both species.

Microsatellites, or short tandem repeats (STR), are biparentally inherited polymorphic nuclear markers that have been developed as one of the most popular genetic markers owing to their high reproducibility, multi-allelic nature, codominant mode of inheritance, abundance and wide genome coverage [52]. Microsatellites are scattered throughout a broad spectrum of prokaryotic and eukaryotic genomes in multiple copies, both in protein-coding and non-coding regions [70] and their density and distribution vary markedly across genomes [16]. For newly analysed taxa, the microsatellites need to be *de novo* characterized and the primers used for their PCR amplification are usually species-specific [70]. Microsatellite markers were originally designed for *D. dendriticus* by library screening using a next-generation sequencing (NGS) approach [10] and have recently been applied in the study of genetic diversity and intercontinental dispersal of temperate and subarctic populations of *D. dendriticus* [31]. Since the primers for STR loci in *D. dendriticus* should be species-specific, their cross-reactivity was tested on *D. ditremus* DNA. The specificity of primers was assessed with the aim of identifying suitable candidates for differentiation between both congeners.

## Materials and methods

### Ethics

Fishes were caught and killed by local professional fishermen and provided to the authors who performed an autopsy and isolated the larvae.

### Parasitic material and its molecular identification

The specificity of primers was tested on 32 tapeworms, namely 16 *D. dendriticus* (Dde) and 16 *D. ditremus* (Ddi) plerocercoids from four localities; lakes Takvatn (NO-TA) and Kalandsvatn (NO-KA) in Norway (NO) and lakes Hafravatn (IS-HA) and Thingvallavatn (IS-TH) in Iceland (IS) (Table 1). Each population of both species was represented by four larvae. The plerocercoids from NO-TA, NO-KA and IS-HA were isolated from the brown trout (*Salmo trutta*) and larvae from IS-TH were obtained from the Arctic char (*Salvelinus alpinus*). The larvae were isolated from capsules localized in the abdominal cavity and on the internal organs of infected fish, rinsed in physiological saline solution, identified by their size and morphology, and preserved in 96% ethanol. Genomic DNA of 32 larvae was isolated using a QIAamp^®^ DNA Mini Kit (QIAGEN, Hilden, Germany), according to the manufacturer’s recommendations. The DNA was stored in deionized water at −20 °C.

**Table 1:** Details on *Dibothriocephalus dendriticus* and *Dibothriocephalus ditremus* specimens used in the current study for tests of specificity of primers.

Country	Locality	Species code	Host	Accession number
*Dibothriocephalus dendriticus* (Dde)				
Norway (NO)	Takvatn (TA)	Dde-NO-TA/11	*Salmo trutta*	OM289894 ^a^
		Dde-NO-TA/12	*S. trutta*	OM289895 ^a^
		Dde-NO-TA/13	*S. trutta*	OM289896 ^a^
		Dde-NO-TA/14	*S. trutta*	OM289897 ^a^
	Kalandsvatn (KA)	Dde-NO-KA/4/1	*S. trutta*	OM289851 ^a^
		Dde-NO-KA/4/2	*S. trutta*	OM283789 ^a^
		Dde-NO-KA/4/3	*S. trutta*	OM286875 ^a^
		Dde-NO-KA/4/4	*S. trutta*	OM289852 ^a^
Iceland (IS)	Hafravatn (HA)	Dde-IS-HA/1/1	*S. trutta*	OM295688 ^a^
		Dde-IS-HA/1/2	*S. trutta*	OM321445 ^a^
		Dde-IS-HA/1/3	*S. trutta*	OM295689 ^a^
		Dde-IS-HA/1/8	*S. trutta*	OM295690 ^a^
	Thingvallavatn (TH)	Dde-IS-TH/2/1	*Salvelinus alpinus*	OM292612 ^a^
		Dde-IS-TH/2/2	*S. alpinus*	OM292613 ^a^
		Dde-IS-TH/2/32	*S. alpinus*	OM321476 ^a^
		Dde-IS-TH/2/33	*S. alpinus*	OM321477 ^a^
*Dibothriocephalus ditremus* (Ddi)				
Norway (NO)	Takvatn (TA)	Ddi-NO-TA/35	*S. trutta*	PV124097 ^b^
		Ddi-NO-TA/36	*S. trutta*	PV124098 ^b^
		Ddi-NO-TA/37	*S. trutta*	PV124099 ^b^
		Ddi-NO-TA/38	*S. trutta*	PV124100 ^b^
	Kalandsvatn (KA)	Ddi-NO-KA/1/1	*S. trutta*	PV124101 ^b^
		Ddi-NO-KA/1/3	*S. trutta*	PV124102 ^b^
		Ddi-NO-KA/1/4	*S. trutta*	PV124103 ^b^
		Ddi-NO-KA/1/5	*S. trutta*	PV124104 ^b^
Iceland (IS)	Hafravatn (HA)	Ddi-IS-HA/3/2	*S. trutta*	PV124105 ^b^
		Ddi-IS-HA/3/3	*S. trutta*	PV124106 ^b^
		Ddi-IS-HA/3/4	*S. trutta*	PV124107 ^b^
		Ddi-IS-HA/3/5	*S. trutta*	PV124108 ^b^
	Thingvallavatn (TH)	Ddi-IS-TH/2/4	*S. alpinus*	PV124109 ^b^
		Ddi-IS-TH/2/5	*S. alpinus*	PV124110 ^b^
		Ddi-IS-TH/2/6	*S. alpinus*	PV124111 ^b^
		Ddi-IS-TH/2/7	*S. alpinus*	PV124112 ^b^

Note: Accession numbers indicate GenBank accession numbers of partial (308 bp) *cox*1 sequences obtained after PCR amplification with *D. dendriticus*-specific primers designed by Wicht *et al.* [66] and used for the exact taxonomic identification of each individual involved in the study. ^a^Sequences published in Králová-Hromadová *et al.* [31]; ^b^Sequences published in the current study.

**Figure 1:**
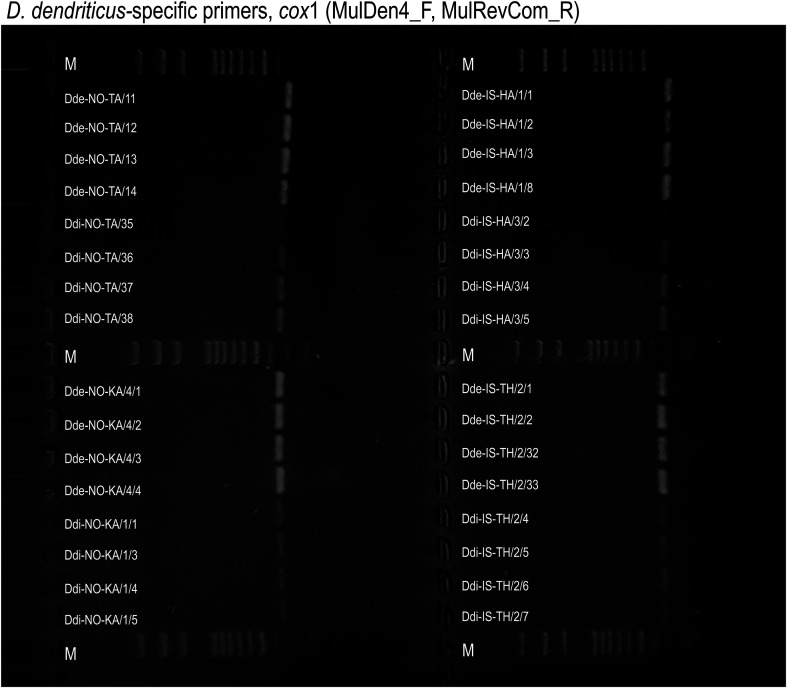
PCR amplification of genomic DNA of *Dibothriocephalus dendriticus* (Dde) and *Dibothriocephalus ditremus* (Ddi) from Takvatn lake, Norway (NO-TA), Kalandsvatn lake, Norway (NO-KA), Hafravatn lake, Iceland (IS-HA) and Thingvallavatn lake, Iceland (IS-TH) using *D. dendriticus*-specific *cox*1 primers designed by Wicht *et al.* (2010). Lane M: 100 bp ladder.

The initial molecular genotyping of all 32 larvae was performed by PCR amplification using the universal reverse primer MulRevCom (5′–ATGATAAGGGAYAGGRGCYCA–3′) and the *D. dendriticus*-specific forward primer MulDen4 (5′–GTGTTTTTCATTTGATGATGACCAGTC–3′) (Table 2), which were designed for the amplification of a 308 bp fragment of the mitochondrial *cox*1 gene specifically in *D. dendriticus* [66]. The PCR was performed in a total volume of 20 μL with 10–20 ng of genomic DNA, 10 pmol of each of the two primers and 1× PCR Master Mix (Thermo Fisher Scientific Inc., Waltham, MA, USA). The PCR amplification conditions were 5 min at 95 °C as an initial denaturation step, followed by 40 cycles of 30 s at 95 °C, 1 min at 60 °C, 1 min at 72 °C, and a final polymerization step of 10 min at 72 °C. The PCR products were visualized on a 1.5% agarose gel. Figure 1 shows that the PCR products were amplified not only in 16 *D. dendriticus* individuals, but also in 16 *D. ditremus* specimens from all four localities (NO-TA, NO-KA, IS-HA and IS-TH). The *D. dendriticus*-specific primers evidently lacked specificity, as a single-step PCR-based discrimination between *D. dendriticus* and *D. ditremus* was not effective and PCR products had to be sequenced to confirm the identity of each individual.

**Table 2:** Details on primers used for tests of their specificity for *Dibothriocephalus dendriticus* and *Dibothriocephalus ditremus.*

Gene	F primer name	F primer sequence (5′–3′)	R primer name	R primer sequence (5′–3′)	PCR product size (bp)	Primer designed as specific for	Specificity −/+	Reference
*cox*1 mtDNA	MulDen4	GTGTTTTTCATTTGATGATGACCAGTC	MulRevCom	ATGATAAGGGAYAGGRGCYCA	~ 310	*D. dendriticus*	−	1
*cox*1 mtDNA	Dde_cox1_F	CAGCCTTTGTTGATAAATT	Dde_cox1_R	AGTGAAACACGACTTTTTAGA	~ 480	*D. dendriticus*	−	2
*cox*1 mtDNA	Ddi_cox1_F	CGGCTTTTGTTGATAATTT	Ddi_cox1_R	AATGAAACACGACTATTTAAA	~ 480	*D. ditremus*	−	2
*cob* mtDNA	Dde_cob_F1	TGTATGTAGCTGACTCAGAATTGA	Dde_cob_R1	ATACTTTCAGCTATAGCAGTTAAAA	~ 290	*D. dendriticus*	−	2
*cob* mtDNA	Ddi_cob_F1	TATATGTGGCAGACTCAGAATTAA	Ddi_cob_R1	ACACTTTCTGCTATTGCAGTTAACA	~ 290	*D. ditremus*	−	2
*cob* mtDNA	Dde_cob_F2	CTATGCTTCGCTCTGTTGAGT	Dde_cob_R2	GCTCTAAAAGTGCTTGATC	~ 270	*D. dendriticus*	−	2
*cob* mtDNA	Ddi_cob_F2	CCATGCTTCGTTCAGTTGAAT	Ddi_cob_R2	GCTCTAGAAATATTAGATC	~ 270	*D. ditremus*	−	2
STR locus Dd_2	Dd_2_F	CCGACAACAACGCTCTAATCC	Dd_2_R	TGCCATTCAGCAAGGTGGAA	~ 210	*D. dendriticus*	−	3
STR locus Dd_8	Dd_8_F	CGTCTATGATCACGCATGTCA	Dd_8_R	CGCTGTAGGATTAGATTCACACG	~ 250	*D. dendriticus*	+	2
STR locus Dd_17	Dd_17_F	ACGCTACTGCATAGATCGAGG	Dd_17_R	GCATAACGCGCCAGAAACAA	~ 240	*D. dendriticus*	−	3
STR locus Dd_23	Dd_23_F	CACACGCAGAAGTCTAGTTGAC	Dd_23_R	TGTTAGCTTACTTCCGTGGCT	~ 140	*D. dendriticus*	−	3
STR locus Dd_25	Dd_25_F	GTTATCCTACGTTGGGCTCCT	Dd_25_R	ATCTGGTTGGGAGAAACAACT	~ 90	*D. dendriticus*	−	3
STR locus Dd_33	Dd_33_F	TGTTTGCTCCAGTGCCTCG	Dd_33_R	CTAGCAGCATCAGCAGTGGA	~ 270	*D. dendriticus*	+	3
STR locus Dd_38	Dd_38_F	ACTATCACGATGCGCTGACA	Dd_38_R	ATCCTTTGTTCCCTGAGCAG	~ 250	*D. dendriticus*	−	3
STR locus Dd_43	Dd_43_F	CAGTCTTTCCGGGTGAAGCT	Dd_43_R	GGTAGCTGCAGTACCGATCA	~ 210	*D. dendriticus*	−	3
STR locus Dd_47	Dd_47_F	ACTTCGGATTACTTCATTAACTCAGT	Dd_47_R	TGGTGAACGAAGTCAAACTATGC	~ 190	*D. dendriticus*	−	3
STR locus Dd_49	Dd_49_F	ACGTCTGACGACAACTTGGG	Dd_49_R	AAGACCCTGGCCAATACACG	~ 190	*D. dendriticus*	−	3
STR locus Dd_57	Dd_57_F	AACATGCGAGTCCCAGGAAG	Dd_57_R	AGCAACGATCTACCGTAAAGCA	~ 120	*D. dendriticus*	−	3
STR locus Dd_78	Dd_78_F	GCTTTCGGCCATTTGTGGTC	Dd_78_R	GGGACAATAGGCAGGGTCTG	~ 270	*D. dendriticus*	−	3
STR locus Dd_84	Dd_84_F	AGAGGTAATTCATCGAGTTCTCTGA	Dd_84_R	TGACTGTGTACATCCGGTCG	~ 240	*D. dendriticus*	−	3
STR locus Dd_95	Dd_95_F	CGTTCACGCTCCAATGATCC	Dd_95_R	AGAGCTTGCTGATGATGGCT	~ 190	*D. dendriticus*	−	3
STR locus Dd_114	Dd_114_F	ACTTCAGGTAATCTCCGTGTCC	Dd_114_R	CTAGCGCCAATGGGTAGCTT	~ 130	*D. dendriticus*	−	3

F, forward primer; R, reverse primer; *cox*1 mtDNA, mitochondrial gene cytochrome *c* oxidase subunit 1; *cob* mtDNA, mitochondrial gene cytochrome b; STR, short tandem repeat (microsatellite); +, specificity confirmed; −, specificity not confirmed; 1, Wicht *et al.* [66]; 2, current study; 3, Bazsalovicsová *et al.* [10].

The PCR products (Fig. 1) were purified using ExoProStar^TM^ 1-Step (Illustra^TM^, GE Healthcare, Little Chalfont, UK), and sequenced from both sides with MulDen4 and MulRevCom primers using a 3500 Genetic Analyzer (Thermo Fisher Scientific) and the BigDye Terminator v3.1 Cycle Sequencing Kit (Thermo Fisher Scientific). Contiguous sequences were assembled and analyzed for errors using Geneious software (version 10.0.5, Biomatters, Auckland, New Zealand). All 16 *D. dendriticus* sequences were compared to the reference *cox*1 sequence of *D. dendriticus* from *Coregonus lavaretus* from Loch Lomond in Scotland, United Kingdom (GU997618). The 16 *D. ditremus* sequences were aligned with the reference sequence of *D. ditremus* from *S. alpinus* from Loch Doyne in Scotland (FM209182). The partial *cox*1 sequences of the 32 *Dibothriocephalus* specimens were deposited in GenBank under the accession numbers indicated in Table 1.

### Data on rRNA and mtDNA genes sequences of *D. dendriticus* and *D. ditremus* from GenBank

Data on the ribosomal and the mitochondrial genes for *D. dendriticus* and *D. ditremus* were retrieved from GenBank (https://www.ncbi.nlm.nih.gov/genbank/). Summary data on the sequences of the small rRNA gene subunit (*ssr*DNA), the large rRNA gene subunit (*lsr*DNA), and the internal transcribed spacers 1 and 2 (ITS1 and ITS2 rDNA) are presented in Supplementary Table 1. Supplementary Table 2 provides data on the sequences of mtDNA genes of *D. dendriticus* and *D. ditremus*. The most frequently studied mitochondrial gene was *cox*1, followed by cytochrome *b* (*cob*). Only a limited number of records were available for subunit 6 of adenosine triphosphatase (*atp*6), subunit 3 of nicotinamide dehydrogenase (*nad*3), and the small (12S) and large (16S) subunits of the mitochondrial rRNA gene.

The sequences were downloaded from GenBank and analyzed using Geneious software and Clustal Omega Multiple Sequence Alignment (https://www.ebi.ac.uk/jdispatcher/msa/clustalo). The analyses were focused on (i) the determination of intraspecific variation of the respective DNA region/gene within both species; (ii) the assessment of interspecific differences between corresponding DNA regions of both species; (iii) the determination of DNA regions suitable for design of species-specific primers, particularly regions with a sufficient level of interspecific differences and, at the same time, without intraspecific variation. Primers designed as potential candidates were tested by PCR amplification using DNA of 16 *D. dendriticus* and 16 *D. ditremus* individuals (Table 1), under the PCR conditions described above.

### Test of specificity of STR primers designed for *D. dendriticus*

The sequences of forward and reverse primers of STR loci designed by Bazsalovicsová *et al.* [10] are listed in Table 2. Since they were specifically designed for *D. dendriticus* and were supposed to be *D. dendriticus*-specific, their specificity was also tested on 16 individuals of *D. ditremus* (Table 1) using the PCR conditions published by Bazsalovicsová *et al.* [10].

## Results

### Interspecific differences of rRNA gene subunits and spacers

**Figure 2:**
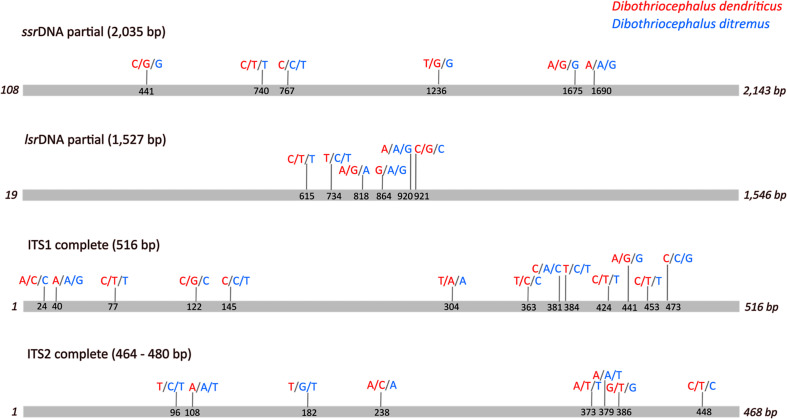
Graphical scheme of mutation sites within the contiguous sequences of the small rRNA gene subunit (*ssr*DNA), the large rRNA gene subunit (*lsr*DNA) and the internal transcribed spacers 1 and 2 (ITS1 and ITS2 rDNA) of *Dibothriocephalus dendriticus* and *Dibothriocephalus ditremus* based on sequences presented in Supplementary Table 1. ITS2 sequences published by Rozas *et al.*, 2012 (468 bp) were used as the reference sequence. The length of ITS2 spacer varied from 464 bp to 480 bp due to different number of repetitive motifs and insertions/deletions.

The determination of interspecific differences and intraspecific variations of *ssr*DNA, *lsr*DNA, ITS1 and ITS2 was based on alignments of all sequences available in GenBank which are summarized in Supplementary Table 1. The alignments resulted in the final contiguous sequences and determination of the mutation sites, which are graphically displayed in Figure 2. The contiguous *ssr*DNA (2,035 bp; partial) and *lsr*DNA (1,527 bp; partial) each contained six non-specific mutations. Sequence alignments of ITS1 resulted in the contiguous 516 bp (complete) ITS1 region with 13 non-specific mutations. The length of the complete ITS2 spacer varied between 464 bp and 480 bp due to the insertions/deletions and the different number of repetitive motifs. The contiguous ITS2 sequence revealed eight non-specific mutations (Fig. 2).

The percentage identity between *D. dendriticus* and *D. ditremus* was high (99.8–100% for *ssr*DNA; 99.7–99.9% for *lsr*DNA; 99.4–100% for ITS1; 96.7–99.6% for ITS2) and similar to the intraspecific sequence identity in all four rRNA gene regions of both species (Table 3).

**Table 3:** Percentage identity of rRNA gene subunits/spacers and mitochondrial genes within and between *Dibothriocephalus dendriticus* and *Dibothriocephalus ditremus.*

DNA region	Length	*D. dendriticus* (% identity)	*D. ditremus* (% identity)	*D. dendriticus/D. ditremus* (% identity)
rRNA gene subunits and spacers				
* ssr*DNA	Partial; 2,024 bp^a^	100	99.9	99.8–100
* lsr*DNA	Partial; 1,413 bp^a^	99.9–100	99.8–100	99.7–99.9
* *ITS1 rDNA	Partial; 505 bp^a^	99.0–100	99.2–100	99.4–100
* *ITS2 rDNA	Complete; 464–480 bp^a, b^	98.3–100	96.3–100	96.7–99.6
Mitochondrial genes				
*cox*1 mtDNA	Complete; 1,566 bp^c^	96.2–100	96.0–96.9	89.7–91.3
* cob* mtDNA	Complete; 1,107 bp^c^	97.3–100	97.0–98.9	89.3–90.9

*ssr*DNA, small subunit of rRNA gene; *lsr*DNA, large subunit of rRNA gene; ITS1, internal transcribed spacer 1; ITS2, internal transcribed spacer 2; *cox*1, cytochrome *c* oxidase subunit 1; *cob*, cytochrome b.

a Supplementary Table 1 indicates sequences (see “+” mark in “% identity” column) that were trimmed to the uniform length and used for calculation of the percentage identity;

b The length of ITS2 spacer varied from 464 bp to 480 bp due to different number of repetitive motifs and insertions/deletions;

c Supplementary Table 2 indicates complete *cox*1 and *cob* sequences (see “+” mark in “% identity” column) which were used for calculation of the percentage identity.

The high homogeneity of ribosomal subunits and spacers between both *Dibothriocephalus* species, the overall low number of mutations in all four rRNA gene regions, and the absence of species-specific mutations did not result in the identification of DNA regions suitable for the design of the species-specific primers.

### Interspecific differences of mtDNA genes

A higher level of interspecific differences between *D. dendriticus* and *D. ditremus* was detected in the complete *cox*1 (1,566 bp) and *cob* (1,107 bp) genes (89.7–91.3% identity for *cox*1; 89.3–90.9% identity for *cob*) (Table 3). The protein coding genes *atp*6 and *nad*3, as well as the ribosomal subunits 12S and 16S were not analyzed, as the number of records for each of them was low (1–2) in both species (see Supplementary Table 2), and the information was insufficient for an assessment of sequence variation within and between species.

A total of 307 mutations, 68 of which were species-specific, were determined in the complete *cox*1 gene (Supplementary Table 3; species-specific mutations are in red and bold). Regions with a higher number of species-specific mutations arranged in close proximity were chosen as suitable regions for the design of *D. dendriticus*-specific (Dde_cox1_F; Dde_cox1_R) and *D. ditremus*-specific (Ddi_cox1_F; Ddi_cox1_R) primers. The position of the primers is graphically displayed in Supplementary Table 3 (colored boxes) and their details are presented in Table 2.

A total of 70 species-specific mutations out of 160 mutations were determined in the complete *cob* gene (Supplementary Table 4; species-specific mutations are in red and bold). Two regions with a higher number of species-specific mutations arranged in close proximity were chosen as suitable targets for the design of two sets of *D. dendriticus*-specific (Dde_cob_F1 + Dde_cob_R1; Dde_cob_F2 + Dde_cob_R2) and two sets of *D. ditremus*-specific primers (Ddi_cob_F1 + Ddi_cob_R1; Ddi_cob_F2 + Ddi_cob_R2). The positions of the primers are graphically displayed in Supplementary Table 4 (colored boxes) and their details are presented in Table 2.

**Supplementary Figure 1:**
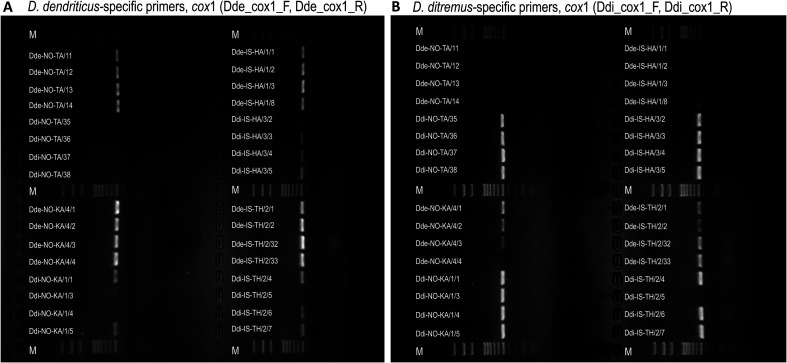
PCR amplification of genomic DNA of *Dibothriocephalus dendriticus* (Dde) and *Dibothriocephalus ditremus* (Ddi) from Takvatn lake, Norway (NO-TA), Kalandsvatn lake, Norway (NO-KA), Hafravatn lake, Iceland (IS-HA) and Thingvallavatn lake, Iceland (IS-TH) using (A) *D. dendriticus*-specific (Dde_cox1_F; Dde_cox1_R) and (B) *D. ditremus*-specific (Ddi_cox1_F; Ddi_cox1_R) *cox*1 primers designed in the current study. Lane M: 100 bp ladder.

**Supplementary Figure 2:**
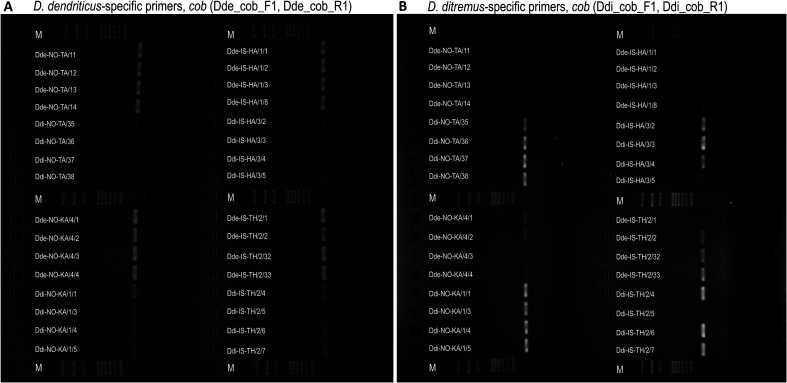
PCR amplification of genomic DNA of *Dibothriocephalus dendriticus* (Dde) and *Dibothriocephalus ditremus* (Ddi) from Takvatn lake, Norway (NO-TA), Kalandsvatn lake, Norway (NO-KA), Hafravatn lake, Iceland (IS-HA) and Thingvallavatn lake, Iceland (IS-TH) using the first set of (A) *D. dendriticus*-specific (Dde_cob_F1; Dde_cob_R1) and (B) *D. ditremus*-specific (Ddi_cob_F1; Ddi_cob_R1) *cob* primers designed in the current study. Lane M: 100 bp ladder.

**Supplementary Figure 3:**
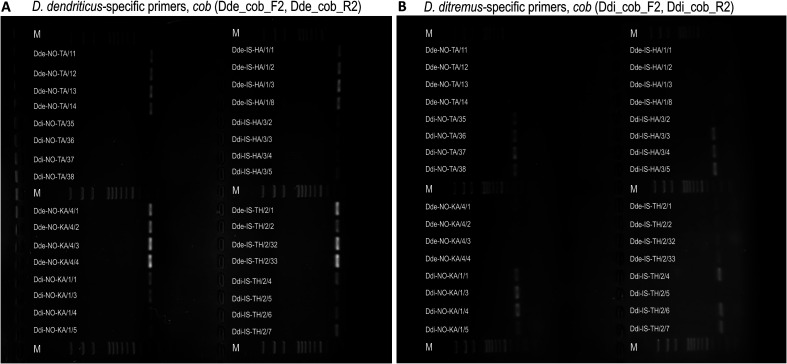
PCR amplification of genomic DNA of *Dibothriocephalus dendriticus* (Dde) and *Dibothriocephalus ditremus* (Ddi) from Takvatn lake, Norway (NO-TA), Kalandsvatn lake, Norway (NO-KA), Hafravatn lake, Iceland (IS-HA) and Thingvallavatn lake, Iceland (IS-TH) using the second set of (A) *D. dendriticus*-specific (Dde_cob_F2; Dde_cob_R2) and (B) *D. ditremus*-specific (Ddi_cob_F2; Ddi_cob_R2) *cob* primers designed in the current study. Lane M: 100 bp ladder.

PCR amplification of 16 *D. dendriticus* and 16 *D. ditremus* individuals with one set of *cox*1 and two sets of *cob D. dendriticus*-specific and *D. ditremus*-specific primers did not confirm their specificity. Similarly, *D. dendriticus*-specific primers also annealed to *D. ditremus* DNA (Supplementary Figs. 1A, 2A, and 3A) and *D. ditremus*-specific primers also provided PCR products on *D. dendriticus* DNA (Supplementary Figs. 1B, 2B, and 3B).

### Specificity of primers for STR loci

**Figure 3:**
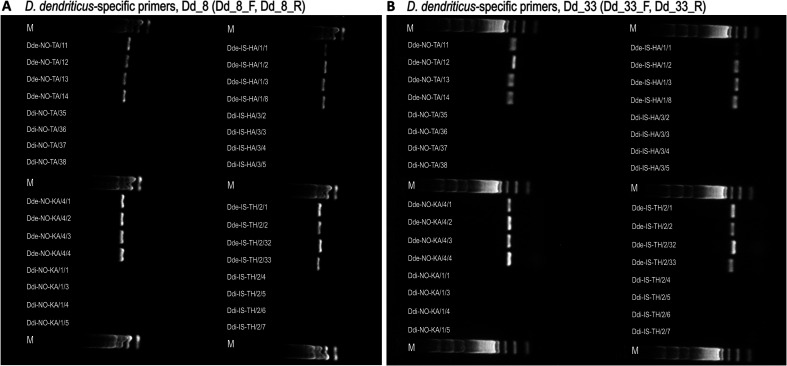
PCR amplification of genomic DNA of *Dibothriocephalus dendriticus* (Dde) and *Dibothriocephalus ditremus* (Ddi) from Takvatn lake, Norway (NO-TA), Kalandsvatn lake, Norway (NO-KA), Hafravatn lake, Iceland (IS-HA) and Thingvallavatn lake, Iceland (IS-TH) using (A) primers amplifying the microsatellite locus Dd_8 and (B) primers amplifying the microsatellite locus Dd_33 designed by Bazsalovicsová *et al.* (2020) for *D. dendriticus*. Lane M: 100 bp ladder.

**Supplementary Figure 4:**
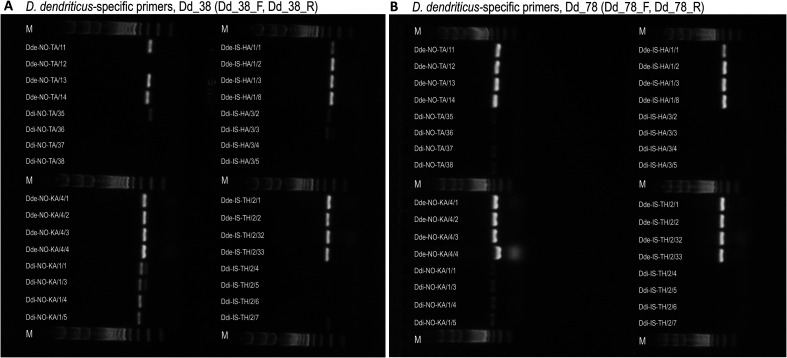
PCR amplification of genomic DNA of *Dibothriocephalus dendriticus* (Dde) and *Dibothriocephalus ditremus* (Ddi) from Takvatn lake, Norway (NO-TA), Kalandsvatn lake, Norway (NO-KA), Hafravatn lake, Iceland (IS-HA) and Thingvallavatn lake, Iceland (IS-TH) using (A) primers amplifying the microsatellite locus Dd_38 and (B) primers amplifying the microsatellite locus Dd_78 designed by Bazsalovicsová et al. (2020) for *D. dendriticus*. Lane M: 100 bp ladder.

Fifteen primer pairs designed for the amplification of STR loci in *D. dendriticus* (Table 2) were tested on DNA from 16 *D. dendriticus* and 16 *D. ditremus* individuals (Table 1). Thirteen primer pairs (namely primers for loci Dd_2, 17, 23, 25, 38, 43, 47, 49, 57, 78, 84, 95, 114; see Table 2 for details) also annealed to DNA of *D. ditremus*, revealing that they are not *D. dendriticus-*specific. The results of PCR amplification with primers amplifying the loci Dd_38 and Dd_78 are presented in Supplementary Figure 4. Two primer sets amplifying the STR loci Dd_8 and Dd_33 proved to be *D. dendriticus*-specific as they provided PCR products exclusively in *D. dendriticus* specimens (Fig. 3).

## Discussion

The current study aimed to develop a species-specific PCR-based method for the single-step discrimination between *D. dendriticus* and *D. ditremus*. Intraspecific variation and interspecific differences were initially analyzed in the small and large subunits and spacers of rRNA genes and protein-coding genes of mitochondrial DNA, highly informative molecular tools in taxonomy, diagnostics, phylogeny, evolution and population genetics [27, 29].

Previous studies revealed that *ssr*DNA and *lsr*DNA are effective markers in phylogenetic studies and in assessing the evolutionary history of diphyllobothriideans [14, 18, 25, 40, 58, 64]. However, the current data showed that *ssr*DNA and *lsr*DNA were not informative enough to distinguish between *D. dendriticus* and *D. ditremus*, as their overall sequences were similar, the number of mutations was low, and none of the mutations were species-specific. Even though the ribosomal spacers ITS1 and ITS2 are frequently used for discrimination at the species and subspecies levels [43], they were found to be unsuitable for detection of differences among diphyllobothriideans [38, 40, 64, 66, 69]. The current study supports the previously published results on the low informativeness of the ribosomal spacers, which were not suitable to distinguish between *D. dendriticus* and *D. ditremus,* due to their similar ITS1 sequence structure, low number of mutations, and an absence of species-specific mutations. Even though the ITS2 spacer was the most variable rRNA gene region, its variability was mainly due to deletions/insertions and a different number of short repetitive regions, which is a rather variable and not reliable parameter for the design of species-specific primers.

Because ribosomal subunits and spacers were not suitable targets for the design of discrimination tools between *D. dendriticus* and *D. ditremus*, mitochondrial DNA, which evolves faster than nuclear genes and is suitable for detection of interrelationships among closely related organisms, was investigated in detail. Previous studies on Diphyllobothriidea showed that mitochondrial *cox*1 is suitable for molecular identification and discrimination of diphyllobothriid tapeworms and assessment of their phylogeny [25, 38, 64, 66, 69]. The present study revealed a high number of mutations, including species-specific ones, in the *cox*1 and *cob* genes. Comparison of the sequences enabled differentiation between *D. dendriticus* and *D. ditremus* because intraspecific sequence variation was lower than the differences between the species. Despite careful selection, primers designed in the *cox*1 and *cob* regions with accumulation of species-specific mutations did not provide the expected results. The designed primer sets also annealed to DNA of the other congener, likely due to unpredictable intraspecific variability in the DNA regions applied for the design of primers.

Since rDNA and mtDNA were not potential candidates for the design of species-specific primers, we were forced to consider other alternatives. Therefore, we decided to test the specificity of 15 recently designed primer pairs for amplification of microsatellite loci in *D. dendriticus* [10]. The specificity of 13 primer pairs was not confirmed, as they also annealed to *D. ditremus* DNA. This was apparently the result of a very similar DNA structure between both congeners especially in the DNA regions used for the design of primers. Two primer sets (for loci Dd_8 and Dd_33) yielded favorable results and amplified the PCR product only in the DNA of *D. dendriticus*. It is evident that DNA regions, which were used as the targets for the design of Dd_8 and Dd_33 primers, displayed sufficient level of interspecific differences.

The latest data have shown that determining the limitations of PCR-based identification methods for diphyllobothriideans is very important. The non-specific amplification of *D. dendriticus*-specific primers developed by Wicht *et al.* [66] on *D. ditremus* DNA ([31]; current study) is justifiable, as these primers were originally designed, tested and validated for four diphyllobothriids infecting humans. Their cross-reactivity with the DNA of *D. ditremus*, a parasite of birds, was not tested and could not have been predicted. However, contradictory interpretations of genotyping with the *cox*1 primers developed by Wicht *et al.* [66] were documented more than a decade ago. While Esteban *et al.* [17] identified a tapeworm from a human patient from Spain as *D. latus,* molecular reassessment by Kuchta *et al.* [34] led to the identification of the tapeworm as *A. pacificus*. It is difficult to determine whether these contradictory results are due to the low specificity of the *cox*1 primers or to technical or human error. Nevertheless, it is evident that molecular studies focused on more populations and species of diphyllobothriids have provided new insights into the intraspecific variability and interspecific differences in mitochondrial genes of this group of cestodes. These findings highlighted either a need to reassess the specificity and effectiveness of existing molecular discrimination tools or to develop and validate new markers.

A broader application of the Dd_8 and Dd_33 primers for discrimination of plerocercoids of other diphyllobothriids infecting salmonids in different continents still needs to be investigated. In Europe, the differentiation between plerocercoids of *D. latus* and *D. dendriticus*/*D. ditremus* has not been considered problematic mainly due to their distinct morphology and characteristic localization in fish [3–5]. Furthermore, the most common hosts of *D. latus* in Europe are European perch *Perca fluviatilis* and ruffe *Gymnocephalus cernua* (Percidae), Northern pike *Esox lucius* (Esocidae), and burbot *Lota lota* (Lotidae), while salmonids have only occasionally been reported as accidental hosts (for a review see [30] and references therein). Consequently, a probability of misidentification of *D. latus* plerocercoids with *D. dendriticus*/*D. ditremus* in Europe is rather low due to the different range of fish hosts, and a substantial decline in the prevalence of *D. latus* in regions where all three species previously co-occurred (*e.g.* Fennoscandia and the Baltic region) [30].

In South America, *D. latus* and *D. dendriticus* have been introduced from the Northern Hemisphere to Patagonia (Argentina and Chile), *e.g.* [54, 68]. As the main fish hosts of *D. latus* do not occur in the Southern Hemisphere, the tapeworm has successfully adapted to salmonids as alternative accessible hosts [35]. Although the co-occurrence of *D. latus* and *D. dendriticus* in salmonids poses a real risk of misidentification, molecular identification of both tapeworms is possible using *D. latus*-specific and *D. dendriticus*-specific *cox*1 primers designed by Wicht *et al.* [66]. The broad applicability of these primers has been demonstrated in recent population-genetic studies of worldwide populations (including those from South America) of *D. latus* [46, 47] and *D. dendriticus* [31]. We tested the specificity and possible cross-reactivity of the currently presented *D. dendriticus*-specific primers Dd_8 and Dd_33 using DNA isolated from *D. latus* from Argentina. Unexpectedly, primers Dd_8 and Dd_33 yielded non-specific products on *D. latus* DNA (data not shown) and they cannot be used to distinguish between *D. latus* and *D. dendriticus* in South America. This suggests that the DNA regions of primers Dd_8 and Dd_33 in *D. dendriticus* are identical (or nearly identical) to the corresponding DNA regions in *D. latus*.

In North America, several *Dibothriocephalus* species, including *D. dendriticus* and *D. ditremus*, utilize salmonids as second intermediate hosts. Additionally, *D. nihonkaiensis*, a parasite of humans and other mammals, and *D. ursi*, parasitizing bears, occur in western North America (Canada and USA) while *D. nihonkaiensis* is also distributed along the northern Pacific coast regions of Asia (for a review see [53]). The sockeye salmon *Oncorhynchus nerka* is an example of a salmonid that serves as a host of more *Dibothriocephalus* species, namely *D. dendriticus*, *D. ditremus*, *D. nihonkaiensis,* and *D. ursi* [2, 12, 42, 44, 45, 53]. Since the mitochondrial *cox*1 primers designed by Wicht *et al.* [66] did not target *D. ditremus* and *D. ursi,* and the STR primers Dd_8 and Dd_33 presented in this study were exclusively validated for discrimination between *D. dendriticus* and *D. ditremus*, a reliable single-step genotyping method for identification of *Dibothriocephalus* larvae from salmonids in North America could be a challenge for future research.

## Conclusions

The genomes of *D. dendriticus* and *D. ditremus* apparently share several similar/identical DNA regions, which makes their molecular PCR-based differentiation difficult. A high genetic similarity may hypothetically result from cross-hybridization and exchange of genetic material between adults of both species caused by common definitive hosts (piscivorous birds), frequent co-infections and a sympatric occurrence. However, more detailed molecular and genetic studies are necessary to confirm this hypothesis. Although *D. dendriticus* and *D. ditremus* could not be discriminated by PCR using *cox*1 and *cob* primers designed in the species-specific regions of both congeners, their differentiation is possible by *cox*1 and *cob* sequence comparisons because interspecific differences of both mitochondrial genes clearly exceeded their intraspecific variation. The current study confirmed previously published findings that rRNA gene subunits and spacers are not suitable for molecular differentiation between diphyllobothriideans. It was surprising that of the 15 primer sets designed for *D. dendriticus* to amplify microsatellite loci, only two proved to be species-specific. The broader application of the primer set for the Dd_8 locus was tested on ~3,500 *D. dendriticus* and *D. ditremus* plerocercoids from different localities in Iceland (318 *D. dendriticus* and 1,366 *D. ditremus*) and Norway (321 *D. dendriticus* and 1,482 *D. ditremus*) (data not shown). The results were confirmed by sequence analyses and showed effectiveness and high reproducibility of the Dd_8 primers, which are recommended for future PCR-based molecular differentiation between *D. dendriticus* and *D. ditremus.*

## Supplementary material

The supplementary material of this article is available at https://www.parasite-journal.org/10.1051/parasite/2025033/olm. *Supplementary Table 1*: Summary data on the sequences of the rRNA gene subunits and spacers of *Dibothriocephalus dendriticus* and *Dibothriocephalus ditremus* retrieved from GenBank. *Supplementary Table 2*: Summary data on the sequences of the mitochondrial DNA genes of *Dibothriocephalus dendriticus* and *Dibothriocephalus ditremus* retrieved from GenBank. *Supplementary Table 3*: Mutation sites (species-specific mutations are in red) and design of the species-specific primers (in colored boxes) in the *cox*1 gene of *Dibothriocephalus dendriticus* and *Dibothriocephalus ditremus*. *Supplementary Table 4*: Mutation sites (species-specific mutations are in red) and design of the species-specific primers (in colored boxes) in the *cob* gene of *Dibothriocephalus dendriticus* and *Dibothriocephalus ditremus*.
