# Supplementary material for: Usefulness of microsatellite loci for differentiating between Dibothriocephalus dendriticus and Dibothriocephalus ditremus (Cestoda: Diphyllobothriidea)
Source: Parasite. 2025 Jul 4;32:41. doi: 10.1051/parasite/2025033 (PMC12232403; doi:10.1051/parasite/2025033)
Supplement: Supplementary file 1 — Supplementary Table 1: Summary data on the sequences of the rRNA gene subunits and spacers of Dibothriocephalus dendriticus and Dibothriocephalus ditremus retrieved from GenBank. [file parasite-32-41-s1.pdf]

Supplementary Table 1. Summary data on the sequences of the rRNA gene subunits and spacers of *Dibothriocephalus dendriticus* and *Dibothriocephalus ditremus* retrieved from GenBank.

rRNA gene subunit/spacer	Length (bp)	GenBank Acc. No.	No. of seqs	% identity	Locality	Host	Reference
Dibotriocephalus dendriticus							
***ssr*DNA** (partial)	2,024	KY552778	1	+	United Kingdom, Loch Lomond	*Coregonus lavaretus* (F)	Waeschenbach et al., 2017
	2,024	KY552779	1	+	USA, Kansas	*Larus hyperboreus* (B)	Waeschenbach et al., 2017
	1,680	AM412739	1		Switzerland	Human	Wicht et al., 2008
	921	DQ181945	1		Norway, Fjellfrøsvatn	*Salvelinus alpinus* (F)	Nicoulaud et al., unpublished
	921	DQ768164	1		Norway, Fjellfrøsvatn	*S. alpinus* (F)	Nicoulaud et al., unpublished
	634	HQ682066	1		Switzerland	Human	de Marval et al., 2013
***lsr*DNA** (partial)	1,516	KR269751	1	+	United Kingdom	*C. lavaretus* (F)	Hernández-Orts et al., 2015
	1,514	KR269757	1	+	Czech Republic	Human	Hernández-Orts et al., 2015
	1,478	KY552814	1	+	USA, Kansas	*L. hyperboreus* (B)	Waeschenbach et al., 2017
	1,478	KY552812	1	+	United Kingdom, Loch Lomond	*C. lavaretus* (F)	Waeschenbach et al., 2017
**ITS1** (complete) + **ITS2** (complete)	516 + 473[b]	FM204787	1	+	Switzerland	Human	Wicht et al., 2008
	516 + 468[a]	JN153006 – JN153015	10	+	Chile, Lake Tarahuín	*Oncorhynchus mykiss* (F)	Rozas et al., 2012
	516 + 468[a]	JN153016 – JN153017	2	+	Chile, Lake Natri	*O. mykiss* (F)	Rozas et al., 2012
	516 + 468[a]	JN153018	1	+	Chile, Lake San Antonío	*O. mykiss* (F)	Rozas et al., 2012
**ITS1** (complete)	516	DQ768177 – DQ768178	2	+	Norway, Fjellfrøsvatn	*S. alpinus* (F)	Nicoulaud et al, unpublished
**ITS1** (partial)	505	Acc. No. 1	18	+	Mongolia, Lake Hövsgöl	*Thymallus nigrescens* (F)	Kutyrev and Mordvinov, 2022
	505	Acc. No. 2	3	+	Mongolia, Lake Hövsgöl	*Brachymystax lenok* (F)	Kutyrev and Mordvinov, 2022
	505	Acc. No. 3	23	+	Russia, Lake Baikal	*Coregonus migratorius* (F)	Kutyrev and Mordvinov, 2022
	504	HQ682065	1		Switzerland	Human	de Marval et al., 2013
	480	JQ245494 – JQ245498	5		Russia, Lake Baikal	*Coregonus autumnalis* (F)	Suleymanov et al., unpublished
**ITS2** (complete)	480[b]	DQ386123	1	+	United Kingdom, Loch Lomond	*C. lavaretus* (F)	Škeříková et al., 2006
	472[b]	DQ386121	1	+	United Kingdom, Loch Lomond	*C. lavaretus* (F)	Škeříková et al., 2006
	466[b]	DQ386122	1	+	United Kingdom, Loch Lomond	*C. lavaretus* (F)	Škeříková et al., 2006
Dibothriocephalus ditremus							
***ssr*DNA** (partial)	2,024	KY552780	1	+	United Kingdom, Loch Doyne	*S. alpinus* (F)	Waeschenbach et al., 2017
	2,023	KY552787	1	+	USA, Oregon, McKenzie River	*Oncorhynchus tshawytscha* (F)	Waeschenbach et al., 2017
	921	DQ181944	1		Norway, Lake Fjellfrøsvatn	*S. alpinus* (F)	Nicoulaud et al., unpublished
	921	DQ768165	1		Norway, Lake Fjellfrøsvatn	*S. alpinus* (F)	Nicoulaud et al., unpublished
***lsr*DNA** (partial)	1,506	KR269750	1	+	United Kingdom	*O. mykiss* (F)	Hernández-Orts et al., 2015
	1,478	KY552813	1	+	United Kingdom, Loch Lomond	*S. alpinus* (F)	Wicht et al., 2010
	1,413	KY552815	1	+	USA, Oregon, McKenzie River	*O. tshawytscha* (F)	Waeschenbach et al., 2017
**ITS1** (complete) + **ITS2** (complete)	516 + 465[b]	DQ768179 – DQ768180	2	+	Norway, Lake Fjellfrøsvatn	*S. alpinus* (F)	Nicoulaud et al., unpublished
**ITS1** (partial)	505	MZ013967 – MZ013968	2	+	Russia, Lake Baikal	*C. migratorius* (F)	Kutyrev and Mordvinov, 2022
	505	MZ014006	1	+	Russia, Lake Baikal	*C. migratorius* (F)	Kutyrev and Mordvinov, 2022
	505	MZ014007 – MZ014019	13	+	Russia, Lake Kapylushi	*Coregonus baunti* (F)	Kutyrev and Mordvinov, 2022

	480	JQ245486 – JQ245491	6		Russia, Tyumen Oblast	*Coregonus sardinella* (F)	Suleymanov et al., unpublished
**ITS2** (complete)	471[b]	DQ386124	1	+	United Kingdom, Isle of Arran	*Salmo salar* (F)	Škeříková et al., 2006
	471[b]	DQ386125	1	+	United Kingdom, Loch Arkaig	*S. salar* (F)	Škeříková et al., 2006
	465[b]	DQ386127	1	+	United Kingdom, Loch Doyne	*S. alpinus* (F)	Škeříková et al., 2006
	465[b]	DQ386128	1	+	United Kingdom, Loch Leven	*O. mykiss* (F)	Škeříková et al., 2006
	464[b]	DQ386126	1	+	United Kingdom, Loch Earn	*Salmo trutta* (F)	Škeříková et al., 2006
	465[b]	AY549515	1	+	United Kingdom, Scotland	*S. alpinus* (F)	Logan et al., 2004
**ITS2** (partial)	452	AY549514	1		United Kingdom, Scotland	*S. trutta* (F)	Logan et al., 2004
	363	AY549507	1		United Kingdom, Scotland	*S. salar* (F)	Logan et al., 2004

***ssr*DNA**, small subunit of rRNA gene; ***lsr*DNA**, large subunit of rRNA gene; **ITS1**, internal transcribed spacer 1; **ITS2**, internal transcribed spacer 2; **Acc.**, Accession; **No.**, number; **seqs**, sequences; **% identity**, percent identity; **+**, sequences trimmed to the uniform length and used for calculation of the percent identity as presented in Table 3; **B**, bird; **F**, fish.

[a], ITS2 sequences published by Rozas et al., 2012 were used as the reference sequence; [b], the length of ITS2 spacer varied from 464 bp to 480 bp due to different number of repetitive motifs and insertions/deletions.

Acc. No. 1, MZ013999 – MZ014005, MZ013990 – MZ013997, MZ013985, MZ013986, MZ013988.

Acc. No. 2, MZ013987, MZ013989, MZ013998.

Acc. No. 3, MZ013960 – MZ013966, MZ013969 – MZ013984.

REFERENCES

Hernández-Orts JS, Scholz T, Brabec J, Kuzmina T, Kuchta R. 2015. High morphological plasticity and global geographical distribution of the Pacific broad tapeworm *Adenocephalus pacificus* (syn. *Diphyllobothrium pacificum*): Molecular and morphological survey. Acta Tropica, 149, 168–178.

Kutyrev IA, Mordvinov VA. 2022. Population genetic structure of diphyllobothriid tapeworms (Cestoda: Diphyllobothriidea) parasitising fish in the Baikal Rift Zone. Diseases of Aquatic Organisms, 148, 113–125.

Logan FJ, Horák A, Štefka J, Aydogdu A, Scholz T. 2004. The phylogeny of diphyllobothriid tapeworms (Cestoda: Pseudophyllidea) based on ITS-2 rDNA sequences. Parasitology Research, 94, 10–15.

Marval de F, Gottstein B, Weber M, Wicht B. 2013. Imported diphyllobothriasis in Switzerland: molecular methods to define a clinical case of *Diphyllobothrium* infection as *Diphyllobothrium dendriticum*, August 2010. Eurosurveillance, 18, 1–6.

Rozas M, Bohle H, Sandoval A, Ildefonso R, Navarrete A, Bustos P. 2012. First molecular identification of *Diphyllobothrium dendriticum* plerocercoids from feral rainbow trout (*Oncorhynchus mykiss*) in Chile. Journal of Parasitology, 98, 1220–1226.

Škeříková A, Brabec J, Kuchta R, Jiménez JA, García HH, Scholz T. 2006. Is the human-infecting *Diphyllobothrium pacificum* a valid species or just a South American population of the holarctic fish broad tapeworm, *D. latum*? American Journal of Tropical Medicine and Hygiene, 75, 307–310.

Waeschenbach A, Brabec J, Scholz T, Littlewood DTJ, Kuchta, R. 2017. The catholic taste of broad tapeworms – multiple routes to human infection. International Journal for Parasitology, 47, 831–843.

Wicht B, de Marval F, Gottstein B, Peduzzi R. 2008. Imported diphyllobothriasis in Switzerland: molecular evidence of *Diphyllobothrium dendriticum* (Nitsch, 1824). Parasitology Research, 102, 201–204.

Wicht B, Yanagida T, Scholz T, Ito A, Jiménez JA, Brabec J. 2010. Inter- and intra-specific characterization of tapeworms of the genus *Diphyllobothrium* (Cestoda: Diphyllobothriidea) from Switzerland, using nuclear and mitochondrial DNA targets. Parasitology International, 59, 35–39.
