# Supplementary material for: Usefulness of microsatellite loci for differentiating between Dibothriocephalus dendriticus and Dibothriocephalus ditremus (Cestoda: Diphyllobothriidea)
Source: Parasite. 2025 Jul 4;32:41. doi: 10.1051/parasite/2025033 (PMC12232403; doi:10.1051/parasite/2025033)
Supplement: Supplementary file 2 — Supplementary Table 2: Summary data on the sequences of the mitochondrial DNA genes of Dibothriocephalus dendriticus and Dibothriocephalus ditremus retrieved from GenBank. [file parasite-32-41-s2.pdf]

Supplementary Table 2. Summary data on the sequences of the mitochondrial DNA genes of *Dibothriocephalus dendriticus* and *Dibothriocephalus ditremus* retrieved from GenBank.

mtDNA gene	Length (bp)	GenBank Acc. No.	No. of seqs	% identity	Locality	Host	Reference
Dibothriocephalus dendriticus							
**mitochondrial genome**	13,715	MW602518	1	+	Czech Republic	Human	Fraija-Fernández et al., 2021
***cox*1** (complete)	1,566	KC812048	1	+	the Netherlands	Human	Kuchta et al., 2013
	1,566	KC812047	1	+	Czech Republic	Human	Kuchta et al., 2013
	1,566	KC812046	1	+	Russia, Lake Baikal	*Coregonus autumnalis* (F)	Kuchta et al., 2013
	1,566	KC812045	1	+	United Kingdom, Loch Lomond	*Coregonus lavaretus* (F)	Kuchta et al., 2013
	1,566	KY552870	1	+	United Kingdom, Loch Doyne	*C. lavaretus* (F)	Waeschenbach et al., 2017
	1,566	AM412738	1	+	Switzerland	Human	Wicht et al., 2008
	1,566	PQ152138	1	+	Greenland, Kobbefjord	*Gasterosteus aculeatus* (F)	Kuchta and Brabec, unpublished
	1,566	PQ152137	1	+	USA, Alaska, Lake Kelly	*Oncorhynchus mykiss* (F)	Kuchta and Brabec, unpublished
	1,566	LC043137 – LC043138	2	+	Chile, Lake Panguipulli	*Salmo trutta* (F)	Yamasaki et al., 2023
	1,566	LC612776	1	+	USA, Kansas	*Larus hyperboreus* (B)	Yamasaki et al., 2023
	1,566	LC609828	1	+	USA, Alaska, Lake Kelly	*O. mykiss* (F)	Yamasaki et al., 2023
	1,566	Acc. No. 1	28	+	Chile, Lake Panguipulli	*O. mykiss* (F)	Yamasaki et al., 2023
	1,566	Acc. No. 2	20	+	Chile, Lake Llanquihue	*Oncorhynchus kisutch* (F)	Yamasaki et al., 2023
	1,566	AB573182	1	+	Russia, Lake Azabachye	*Salvelinus leucomaenis* (F)	Yamasaki et al., 2023
	1,566	Acc. No. 3	3	+	Chile, Lake Llanquihue	*O. kisutch* (F)	Yamasaki et al., 2023
	1,566	AB623150	1	+	Chile, Los Lagos region	*O. kisutch* (F)	Yamasaki et al., 2023
	1,566	MZ603738 – MZ603741	4	+	Chile, Lake Panguipulli	*Oncorhynchus tshawytscha* (F)	Rubilar et al., 2022
***cox*1** (partial)	711	AB374223	1		Russia, Lake Azabachye	*S. leucomaenis* (F)	Arizono et al., 2009
	678	MW979692 – MW979714	23		Russia, Lake Baikal	*Coregonus migratorius* (F)	Kutyrev and Mordvinov, 2022
	678	Acc. No. 4	14		Mongolia, Lake Hövsgöl	*Thymallus nigrescens* (F)	Kutyrev and Mordvinov, 2022
	678	Acc. No. 5	4		Mongolia, Lake Hövsgöl	*Brachymystax lenok* (F)	Kutyrev and Mordvinov, 2022
	599	KC812049	1		USA, Kansas	*L. hyperboreus* (B)	Kuchta et al., 2013
	574	JN152993 – JN153002	10		Chile, Lake Tarahuín	*O. mykiss* (F)	Rozas et al., 2012
	574	JN153003 – JN153004	2		Chile, Lake Natri	*O. mykiss* (F)	Rozas et al., 2012
	574	JN153005	1		Chile, Lake San Antonío	*O. mykiss* (F)	Rozas et al., 2012
	550	JQ245479 – JQ245481	3		Russia, Lake Baikal	*C. autumnalis* (F)	Suleymanov et al., unpublished
	444	MW283886 – MW283887	2		Chile, Lake Panguipulli	*O. tshawytscha* (F)	Rubilar et al., 2022
	398	KJ026490	1		Canada, BC, Lake Owikeno	*Ursus arctos horribilis* (M)	Catalano et al., 2015
	396	DQ768193 – DQ768194	2		Norway, Lake Fjellfrøsvatn	*Salvelinus alpinus* (F)	Yera et al, 2008
	375	HQ682067	1		Switzerland	Human	de Marval et al., 2013
	310	GU997617	1		United Kingdom, Loch Lomond	*C. lavaretus* (F)	Wicht et al., 2010
	308	GU997618	1		United Kingdom, Loch Lomond	*C. lavaretus* (F)	Wicht et al., 2010
	308	MN326445 – MN326452	8		Argentina, Lago Gutiérrez	*O. mykiss* (F)	Kuchta et al., 2019

	308	MN326453 – MN326454	2		Argentina, Lago Gutiérrez	*S. trutta* (F)	Kuchta et al., 2019
	308	MN326455 – MN326456	2		Argentina, Lago Gutiérrez	*Salvelinus fontinalis* (F)	Kuchta et al., 2019
	308	Acc. No. 6	34		Norway, Lake Takvatn	*S. trutta* (F)	Králová-Hromadová et al., 2025
	308	Acc. No. 7	72		Norway, Lake Kalandsvatn	*S. trutta* (F)	Králová-Hromadová et al., 2025
	308	Acc. No. 8	41		Norway, Lake Kalandsvatn	*S. alpinus* (F)	Králová-Hromadová et al., 2025
	308	Acc. No. 9	39		Finland, Ylöjärvi	*Lynx lynx* (M)	Králová-Hromadová et al., 2025
	308	OM289842 – OM289845	4		United Kingdom, Loch Arkaig	*Salmo salar* (F)	Králová-Hromadová et al., 2025
	308	Acc. No. 10	3		United Kingdom, Loch Leven	*O. mykiss* (F)	Králová-Hromadová et al., 2025
	308	Acc. No. 11	17		United Kingdom, Loch Lomond	*C. lavaretus* (F)	Králová-Hromadová et al., 2025
	308	Acc. No. 12	8		United Kingdom, Loch Earn	*S. trutta* (F)	Králová-Hromadová et al., 2025
	308	Acc. No. 13	6		United Kingdom, Loch Earn	*O. mykiss* (F)	Králová-Hromadová et al., 2025
	308	Acc. No. 14	26		Iceland, Lake Hafravatn	*S. trutta* (F)	Králová-Hromadová et al., 2025
	308	Acc. No. 15	4		Iceland, Lake Hafravatn	*S. alpinus* (F)	Králová-Hromadová et al., 2025
	308	Acc. No. 16	229		Iceland, Lake Thingvallavatn	*S. alpinus* (F)	Králová-Hromadová et al., 2025
	308	Acc. No. 17	54		Iceland, Lake Másvatn	*S. trutta* (F)	Králová-Hromadová et al., 2025
	308	Acc. No. 18	5		Iceland, Lake Ytra-Hólavatn	*S. alpinus* (F)	Králová-Hromadová et al., 2025
	308	OM327632 – OM327634	3		Greenland, Lake Amitsorsuaq	*S. alpinus* (F)	Králová-Hromadová et al., 2025
	308	Acc. No. 19	10		USA, Alaska, Lake Kelly	*O. mykiss* (F)	Králová-Hromadová et al., 2025
	308	Acc. No. 20	3		USA, Alaska, Lake West Beaver	*O. mykiss* (F)	Králová-Hromadová et al., 2025
	308	OM327690 – OM327697	8		USA, Alaska, Lake Clunie	*S. alpinus* (F)	Králová-Hromadová et al., 2025
	308	OM327698, OM327719	2		USA, Oregon, Hills Creek Reservoir	*O. mykiss* (F)	Králová-Hromadová et al., 2025
	308	OM327717 – OM327718	2		USA, Oregon, Lake Detroit	*O. mykiss* (F)	Králová-Hromadová et al., 2025
	308	OM372679 – OM372681	3		Argentina, Los Lagos region	*O. mykiss* (F)	Králová-Hromadová et al., 2025
	308	Acc. No. 21	3		Russia, Lake Baikal	*C. autumnalis* (F)	Králová-Hromadová et al., 2025
	296	GU997619	1		Russia, Olkhon Island	*C. autumnalis* (F)	Wicht et al., 2010
	295	GU997616	1		Estonia, Lake Peipsi	*C. lavaretus* (F)	Wicht et al., 2010
	271	MT784739, MT784742	2		Argentina, Lake Moreno	*Galaxias maculatus* (F)	Semenas et al., 2021
	271	MT784740	1		Argentina, Lake Nahuel Huapí	*G. maculatus* (F)	Semenas et al., 2021
	271	MT784738	1		Argentina, Lake Nahuel Huapí	*O. mykiss* (F)	Semenas et al., 2021
	271	MT784741	1		Argentina, Lake Gutiérrez	*O. mykiss* (F)	Semenas et al., 2021
cob (complete)	1,107	LC609830 – LC609842	13	+	Chile, Lake Llanquihue	*O. kisutch* (F)	Yamasaki et al., 2023
	1,107	LC609843 – LC609853	11	+	Chile, Lake Panguipulli	*O. mykiss* (F)	Yamasaki et al., 2023
	1,107	LC609854	1	+	USA, Kansas	*L. hyperboreus* (B)	Yamasaki et al., 2023
	1,107	LC609855	1	+	USA, Lake Kelly	*O. mykiss* (F)	Yamasaki et al., 2023
	1,107	LC742383 – LC742385	3	+	Chile, Lake Llanquihue	*O. kisutch* (F)	Yamasaki et al., 2023
	1,107	LC742386 – LC742388	3	+	Chile, Lake Panguipulli	*O. mykiss* (F)	Yamasaki et al., 2023
	1,107	LC742389	1	+	Russia, Lake Azabachye	*S. leucomaenis* (F)	Yamasaki et al., 2023
	1,107	LC764366	1	+	Chile, Lake Panguipulli	*O. mykiss* (F)	Yamasaki et al., 2023
	1,107	AB522613	1	+	Switzerland, Bern	Human	Wicht et al., 2010

	1,107	AB522614	1	+	Estonia, Lake Peipsi	*C. lavaretus* (F)	Wicht et al., 2010
atp6 (complete)	510	AB516958	1		Russia, Lake Azabachye	*S. leucomaenis* (F)	Yamasaki et al., unpublished
	510	JN040538	1		n. i.	n. i.	Bohle and Gabaldón, 2012
***nad*3** (partial)	357	AB374224	1		Russia, Lake Azabachye	*S. leucomaenis* (F)	Arizono et al., 2009
**16S rRNA** (partial)	967	KY552847	1		United Kingdom, Loch Lomond	*C. lavaretus* (F)	Waeschenbach et al., 2017
	849	KY552848	1		USA, Kansas	*L. hyperboreus* (B)	Waeschenbach et al., 2017
Dibothriocephalus ditremus							
***cox*1** (complete)	1,566	AB979518	1	+	Japan, Hokkaido	*Hypomesus pretiosus japonicus* (F)	Banzai-Umehara et al., 2016
	1,566	FM209182	1	+	United Kingdom, Loch Doyne	*S. alpinus* (F)	Wicht et al., 2010
	1,566	KY552872	1	+	USA, Oregon, McKenzie River	*O. tshawytscha* (F)	Waeschenbach et al., 2017
***cox*1** (partial)	678	MW979733	1		Russia, Lake Baikal	*Coregonus migratorius* (F)	Kutyrev and Mordvinov, 2022
	678	MW979734 – MW979749	16		Russia, Lake Kapylushi	*Coregonus baunti* (F)	Kutyrev and Mordvinov, 2022
	550	JQ245472 – JQ245476	5		Russia, Tyumen Oblast	*Coregonus sardinella* (F)	Suleymanov et al., unpublished
	428	AB437938	1		Japan, Osaka	*Hypomesus transpacificus nipponensis* (F)	Abe, 2009
	396	DQ768195 – DQ768196	2		Norway, Lake Fjellfrøsvatn	*S. alpinus* (F)	Nicoulaud et al., unpublished
cob (complete)	1,107	AB522617	1	+	Finland, Oulu	*Coregonus widegreni* (F)	Wicht et al., 2010
	1,107	AB522618	1	+	United Kingdom, Loch Lomond	*S. alpinus* (F)	Wicht et al., 2010
	1,107	AB979525	1	+	Japan, Hokkaido	*H. pretiosus japonicus* (F)	Banzai-Umehara et al., 2016
atp6 (complete)	510	AB516959	1		Russia, Lake Azabachye	*Salvelinus albus* (F)	Yamasaki et al., unpublished
	510	JN040539	1		n. i.	n. i.	Bohle and Gabaldón, 2012
**16S rRNA** (partial)	808	KY552850	1		United Kingdom, Loch Doyne	*S. alpinus* (F)	Waeschenbach et al., 2017
	381	AB020406	1		Japan	n. i.	Nakao, unpublished
**12S rRNA** (partial)	347	AB031366	1		Japan	n. i.	Nakao, unpublished

cox*1**, cytochrome *c* oxidase subunit 1; ***cob, cytochrome b; ***atp6***, adenosine triphosphatase subunit 6; ***nad*3**, nicotinamide dehydrogenase subunit 3; **16S rRNA**, large subunit of mitochondrial rRNA gene; **12S rRNA**, small subunit of mitochondrial rRNA gene; **Acc.**, Accession; **No.**, number; **seqs**, sequences; **% identity**, percent identity; **+**, sequences used for calculation of the percent identity as presented in Table 3; **B**, bird; **F**, fish; **M**, mammal; **n. i.**, not indicated. **Acc. No. 1**, LC609820 – LC609827, LC609799 – LC609818; **Acc. No. 2**, LC609819, LC609780 – LC609798; **Acc. No. 3**, AB530455 – AB530456, AB623149; **Acc. No. 4**, MW979715 – MW979716, MW979718, MW979721– MW979727, MW979729 – MW979732; **Acc. No. 5**, MW979717, MW979719 – MW979720, MW979728; **Acc. No. 6**, OM283720, OM289885 – OM289917; **Acc. No. 7**, OM283758 – OM283767, OM283771 – OM283774, OM283780 – OM283784, OM283788 – OM283793, OM283800 – OM283804, OM283810 – OM283812, OM286875 – OM286879, OM286738 – OM286746, OM286748, OM286750 – OM286751, OM289846 – OM289861, OM289872 – OM289874, OM289883 – OM289884, OM287397; **Acc. No. 8**, OM283768 – OM283770, OM283775 – OM283779, OM283794 – OM283799, OM283805 – OM283809, OM286747, OM286749, OM286880, OM289862 – OM289871, OM289875 – OM289882, OM292593; **Acc. No. 9**, OM292594 – OM292603, OM303058 – OM303084, OM289918, OM321679; **Acc. No. 10**, OM283721 – OM283722, OM292604; **Acc. No. 11**, OM321600 – OM321606, OM283723 – OM283732; **Acc. No. 12**, OM292605 – OM292606, OM283733, OM283813, OM289944 – OM289947; **Acc. No. 13**, OM289939 – OM289943, OM321607; **Acc. No. 14**, OM295688 – OM295699, OM321445 – OM321455, OM327611 – OM327612, OM327624; **Acc. No. 15**, OM295700 – OM295701, OM321456, OM327625; **Acc. No. 16**, OM292612 – OM292769, OM321476 – OM321543, OM327630; **Acc. No. 17**, OM292770 – OM292798, OM321460 – OM321475, OM327613 – OM327620; **Acc. No. 18**, OM292607 – OM292608, OM321457 – OM321459; **Acc. No. 19**, OM327682 – OM327687, OM327699 – OM327700, OM327714 – OM327716; **Acc. No. 20**, OM327688 – OM327689, OM327715; **Acc. No. 21**, OM292609 – OM292611, OM327681.

REFERENCES

Abe N. 2009. Identification of the larval cestode plerocercoid found in the body cavities of the Japanese smelt *Hypomesus transpacificus nipponensis*, and review of the literature. Seikatsu Eisei, 53, 110–116.

Arizono N, Shedko M, Yamada M, Uchikawa R, Tegoshi T, Takeda K, Hashimoto K. 2009. Mitochondrial DNA divergence in populations of tapeworm *Diphyllobothrium nihonkaiense* and its phylogenetic relationship with *Diphyllobothrium klebanovskii*. Parasitology International, 58, 22–28.

Banzai-Umehara A, Suzuki M, Akiyama T, Ooi H-K, Kawakami Y. 2016. Invalidation of *Diphyllobothrium hottai* (Cestoda: Diphyllobothriidae) based on morphological and molecular phylogenetic analyses. Parasitology International, 65, 459–462.

Bohle HM, Gabaldón T. 2012. Selection of marker genes using whole-genome DNA polymorphism analysis. Evolutionary Bioinformatics, 8, 161–169.

Catalano S, Lejeune M, Tizzani P, Verocai GG, Schwantje H, Nelson C, Duignan PJ. 2015. Helminths of grizzly bears (*Ursus arctos*) and American black bears (*Ursus americanus*) in Alberta and British Columbia, Canada. Canadian Journal of Zoology, 93, 765–772.

Fraija-Fernández N, Waeschenbach A, Briscoe A, Hocking S, Kuchta R, Nyman T, Littlewood DTJ. 2021. Evolutionary transitions in broad tapeworms (Cestoda: Diphyllobothriidea) revealed by mitogenome and nuclear ribosomal operon phylogenetics. Molecular Phylogenetics and Evolution, 163, 107262.

Králová-Hromadová I, Čisovská Bazsalovicsová E, Radačovská A, Šoltys K, Juhásová L, Minárik G, Kuchta R, Skírnisson K, Karlsbakk E, Štefka J. 2025. Genetic diversity and intercontinental dispersal of temperate and subarctic populations of *Dibothriocephalus dendriticus* (Cestoda; Diphyllobothriidea), a causative agent of dibothriocephalosis, International Journal for Parasitology, 55, 281-298.

Kuchta R, Brabec J, Kubáčková P, Scholz T. 2013. Tapeworm *Diphyllobothrium dendriticum* (Cestoda) – neglected or emerging human parasite? PLOS Neglected Tropical Diseases, 7, 1–8.

Kuchta R, Radačovská A, Bazsalovicsová E, Viozzi G, Semenas L, Arbetman M, Scholz T. 2019. Host switching of zoonotic broad fish tapeworm (*Dibothriocephalus latus*) to salmonids, Patagonia. Emerging Infectious Diseases, 25, 2156–2158.

Kutyrev IA, Mordvinov VA. 2022. Population genetic structure of diphyllobothriid tapeworms (Cestoda: Diphyllobothriidea) parasitising fish in the Baikal Rift Zone. Diseases of Aquatic Organisms, 148, 113–125.

Marval de F, Gottstein B, Weber M, Wicht B. 2013. Imported diphyllobothriasis in Switzerland: molecular methods to define a clinical case of *Diphyllobothrium* infection as *Diphyllobothrium dendriticum*, August 2010. Eurosurveillance, 18, 1–6.

Rozas M, Bohle H, Sandoval A, Ildefonso R, Navarrete A, Bustos P. 2012. First molecular identification of *Diphyllobothrium dendriticum* plerocercoids from feral rainbow trout (*Oncorhynchus mykiss*) in Chile. Journal of Parasitology, 98, 1220–1226.

Rubilar A, Torres P, Yera H, Leyan V, Silva R. 2022. First report of zoonotic tapeworms, *Dibothriocephalus latus* (Linnaeus, 1758) and *D. dendriticus* (Nitzsch, 1824), and other endohelminth parasites in chinook salmon, *Oncorhynchus tshawytscha*, in Chile. Comparative Parasitology, 89, 35–54.

Semenas L, Viozzi G, Arbetman M. 2021. A regional study of the zoonotic broad tapeworm *Dibothriocephalus* spp. in Northwestern Patagonia (Argentina): origin of fishes and coastal cities as factors affecting infection in fishes. Parasitology Research, 120, 2415–2427.

Waeschenbach A, Brabec J, Scholz T, Littlewood DTJ, Kuchta, R. 2017. The catholic taste of broad tapeworms – multiple routes to human infection. International Journal for Parasitology, 47, 831–843.

Wicht B, de Marval F, Gottstein B, Peduzzi R. 2008. Imported diphyllobothriasis in Switzerland: molecular evidence of *Diphyllobothrium dendriticum* (Nitsch, 1824). Parasitology Research, 102, 201–204.

Wicht B, Yanagida T, Scholz T, Ito A, Jiménez JA, Brabec J. 2010. Inter- and intra-specific characterization of tapeworms of the genus *Diphyllobothrium* (Cestoda: Diphyllobothriidea) from Switzerland, using nuclear and mitochondrial DNA targets. Parasitology International, 59, 35–39.

Yamasaki H, Sato MO, Kuramochi T, Mercado, R. 2023. Genetic characterization of *Dibothriocephalus latus* and *Dibothriocephalus dendriticus* (Cestoda: Diphyllobothriidae) from Chile based on haplotype analysis using mitochondrial DNA markers. Parasitology International, 96, 102767.

Yera H, Nicoulaud J, Dupoy-Camet J. 2008. Use of nuclear and mitochondrial DNA PCR and sequencing for molecular identification of *Diphyllobothrium* isolates potentially infective for humans. Parasite, 15, 402–407.
