# Supplementary material for: Usefulness of microsatellite loci for differentiating between Dibothriocephalus dendriticus and Dibothriocephalus ditremus (Cestoda: Diphyllobothriidea)
Source: Parasite. 2025 Jul 4;32:41. doi: 10.1051/parasite/2025033 (PMC12232403; doi:10.1051/parasite/2025033)
Supplement: Supplementary file 3 — Supplementary Table 3: Mutation sites (species-specific mutations are in red) and design of the species-specific primers (in colored boxes) in the cox1 gene of Dibothriocephalus dendriticus and Dibothriocephalus ditremus. [file parasite-32-41-s3.pdf]

Supplementary Table 3. Mutation sites (species-specific mutations are in red) and design of the species-specific primers (in coloured boxes) in the *cox*1 gene of *Dibothriocephalus dendriticus* and *Dibothriocephalus ditremus.*

No. of mutation	Position of mutation	Nucleotide in *D. dendriticus*	Nucleotide in *D. ditremus*
1	10	C/T	C
2	13	A/C	A
3	15	A/G/T	A
4	19	G/T	T
5	20	C/T	T
6	36	C/T	T
7	42	C/T	T
8	45	C/T	T
9	66	C/T	T
10	70	C/T	T
11	78	C/T	T
12	87	C/T	C
13	90	A/G	T
14	93	C/T	T
15	96	C/T	C/T
16	105	**T**	**A**
17	111	G/T	T
18	112	G	A/G
19	154	A/G	A
20	163	G	A/G
21	183	**T**	**C**
22	189	C/T	C/T
23	192	**C**	**T**
24	210	**C**	**T**
25	213	C	C/T
26	225	C/T	C
27	228	C/T	T
28	234	**T**	**A**
29	237	G/T	G
30	240	G/T	T
31	261	**C**	**T**
32	265	**T**	**C**
33	270	A/T	A/T
34	272	A/G	G
35	276	**C**	**T**
36	277	C/T	C
37	280	G	A/G
38	288	**T**	**C**
39	291	G	A/G
40	297	**C**	**T**
41	312	T	C/T
42	315	**T**	**G**
43	318	A/G	G
44	322	**T**	**C**
45	324	A/G	A
46	327	T	C/T
47	333	T	A/C/T
48	336	**C**	**A**
49	337	C/T	C
50	339	G	A/G
51	340	G/T	T
52	342	C/T	T
53	348	A/T	C/T
54	353	G	A/G
55	354	A	A/G
56	360	**T**	**C**
57	363	A/C	T
58	366	C/T	T
59	372	A/G	G
60	375	**A**	**T**
61	381	**G**	**A**
62	399	**G**	**A**
63	411	**A**	**G**
64	414	C/T	C/T
65	417	A	A/G
66	422	A/C/G	A/G

67	423	**T**	**A**	
68	432	T	C/T	
69	453	A/G	A	
70	462	T	G/T	
71	466	G/T	T	
72	474	**A**	**G**	
73	477	T	C/T	
74	483	**A**	**T**	
75	504	C/T	G/T	
76	510	C/T	C/T	
77	513	**A**	**G**	**FORWARD PRIMER**
78	516	**C**	**T**	*D. dendriticus*-specific: Dde_cox1_F
79	528	**A**	**T**	*D. ditremus*-specific: Ddi_cox1_F
80	534	C	C/T	
81	537	C/T	C/T	
82	540	**C**	**T**	
83	542	G	A/G	
84	543	T	A/G	
85	553	A/T	T	
86	561	T	A/T	
87	565	C/T	T	
88	570	C/T	T	
89	573	T	C/T	
90	576	A/G	A	
91	603	G/T	G	
92	612	C/T	T	
93	627	**G**	**A**	
94	628	T	C/T	
95	633	T	C/T	
96	651	A	A/G	
97	666	**T**	**A**	
98	667	C/T	T	
99	670	C/G	G	
100	672	C/T	C/T	
101	675	T	C/T	
102	678	A/G	A/T	
103	679	C/G	G	
104	684	C/T	T	
105	693	C/T	T	
106	696	C/G	G	
107	699	C/T	T	
108	708	A/G	A	
109	711	C/T	T	
110	714	C/T	T	
111	717	C/G/T	G/T	
112	720	C/T	C/T	
113	723	C/T	T	
114	726	G	A/G	
115	729	A/G/T	G/T	
116	735	C/T	C/G	
117	741	C/T	T	
118	747	C/T	G	
119	750	**T**	**G**	
120	756	A/G	T	
121	758	C/T	T	
122	765	C/T	C/T	
123	770	C/T	T	
124	780	C/T	T	
125	783	**A**	**G**	
126	786	T	C/T	
127	789	**T**	**C**	
128	792	A/C/T	A	
129	798	C/T	C/T	
130	801	C	C/T	
131	807	A/G	G	
132	816	A/G	G	
133	819	A/G	A/G	
134	822	A	A/G/T	
135	828	C/T	C/T	
136	834	C/T	T	
137	837	C/T	T	

138	840	A	A/T	
139	843	C/T	T	
140	846	C/T	T	
141	847	T	C/T	
142	849	**G**	**A**	
143	850	C/G	G	
144	855	T	C/T	
145	861	**G**	**A**	
146	864	G	G/A	
147	867	G	G/A	
148	870	**T**	**C**	
149	882	**A**	**G**	
150	885	A/C	G	
151	886	C/G	G	
152	889	C/T	T	
153	902	A/C	C	
154	906	**T**	**C**	
155	908	G/T	T	
156	912	**C**	**T**	
157	915	C/T	T	
158	918	C/T	C/T	
159	921	**T**	**A**	
160	924	C/T	A/C/T	
161	925	A/C	A	
162	930	G	A/G	
163	933	C/T	T	
164	942	**T**	**G**	
165	945	**T**	**A**	
166	948	C/T	A/T	
167	966	A/G/T	G	
168	969	A/G	A/G	
169	970	A/C/T	T	
170	972	A	A/G	
171	975	C/T	C/T	
172	982	**C**	**T**	**REVERSE PRIMER**
173	987	**A**	**T**	*D. dendriticus*-specific: Dde_cox1_R
174	1000	**C**	**T**	*D. ditremus*-specific: Ddi_cox1_R
175	1002	C/T	G	
176	1005	G/T	T	
177	1008	G	A/G	
178	1011	**T**	**A**	
179	1014	A/C/T	T	
180	1018	G/T	T	
181	1021	C/T	T	
182	1023	**A**	**G**	
183	1024	A/G	G	
184	1026	T	C/T	
185	1041	G/T	G	
186	1042	T	C/T	
187	1044	A/G	A/G	
188	1053	T	C/T	
189	1056	**G**	**T**	
200	1065	A/G	G	
201	1068	C/T	T	
202	1069	A/G	A	
203	1083	T	C/T	
204	1089	**G**	**T**	
205	1095	C/T	T	
206	1101	T	C/T	
207	1107	T	C/T	
208	1110	C/T	C/T	
209	1116	A	A/G	
210	1119	C/T	T	
211	1122	**T**	**G**	
212	1125	A	A/G	
213	1126	C/G	G	
214	1131	**C**	**T**	
215	1143	A/T	A/T	
216	1144	A/C	A/C	
217	1146	G/T	G	
218	1149	A/G/T	T	

219	1152	A/G	A
220	1155	T	C/T
221	1158	T	C/T
222	1161	C/T	C
223	1164	**A**	**T**
224	1166	A/G	G
225	1168	A	A/G
226	1174	A/G	G
227	1176	A/G	A/G
228	1177	A/T	T
229	1179	T	C/T
230	1182	C/T	T
231	1194	A/G	A
232	1197	A	A/G
233	1200	**C**	**T**
234	1203	T	C/T
235	1206	A/G	A
236	1209	T	A/T
237	1212	A	A/G
238	1215	**A**	**T**
239	1216	T	C/T
240	1224	C/G	G
241	1230	A	A/G
242	1231	C/T	T
243	1236	**A**	**G**
244	1239	C/T	C/T
245	1246	A/G	A
246	1251	**T**	**A**
247	1263	C/G/T	A
248	1266	**T**	**C**
249	1269	A/T	T
250	1270	C/T	T
251	1284	C/T	T
252	1291	A/T	T
253	1293	T	C/T
254	1299	C/T	A/G
255	1300	**G**	**A**
256	1305	C/T	C/T
257	1308	T	C/T
258	1314	A/G	A
259	1326	**C**	**T**
260	1329	A	A/G
261	1332	T	C/T
262	1335	G	A/G
263	1337	C/T	C
264	1338	A/G/T	A
265	1339	A/G	G
266	1351	A/T	A/T
267	1353	C/T	T
268	1359	T	A/G
269	1365	**C**	**T**
270	1368	A	A/T
271	1377	**C**	**T**
272	1386	**T**	**C**
273	1389	C/T	C
274	1395	C/G/T	G
275	1398	T	C/T
276	1404	**T**	**C**
277	1407	A/C	T
278	1410	G	A/G
279	1412	C/T	T
280	1413	C/T	T
281	1422	G	A/G
282	1425	**G**	**A**
283	1428	A	A/C
284	1431	A	A/G
285	1434	**G**	**A**
286	1440	G/T	G
287	1455	T	C/T
288	1458	T	C/T
289	1464	C/T	A

290	1467	C/T	T
291	1468	C/T	T
292	1472	C/G	C
293	1488	A/G	G
294	1491	C	C/T
295	1494	**A**	**G**
296	1501	A/G	G
297	1503	**C**	**T**
298	1506	C/T	T
299	1521	C	C/T
300	1527	**T**	**C**
301	1530	**A**	**G**
302	1533	T	A/G
303	1539	C/T	C/T
304	1540	**A**	**G**
305	1542	A	A/G
306	1548	**T**	**C**
307	1560	C/T	C/T
