# Supplementary material for: Usefulness of microsatellite loci for differentiating between Dibothriocephalus dendriticus and Dibothriocephalus ditremus (Cestoda: Diphyllobothriidea)
Source: Parasite. 2025 Jul 4;32:41. doi: 10.1051/parasite/2025033 (PMC12232403; doi:10.1051/parasite/2025033)
Supplement: Supplementary file 4 — Supplementary Table 4: Mutation sites (species-specific mutations are in red) and design of the species-specific primers (in colored boxes) in the cob gene of Dibothriocephalus dendriticus and Dibothriocephalus ditremus. [file parasite-32-41-s4.pdf]

**Supplementary Table 4.** Mutation sites (species-specific mutations are in red) and design of the species-specific primers (in coloured boxes) in the *cob* gene of *Dibothriocephalus dendriticus* and *Dibothriocephalus ditremus.*

| No. of mutation | Position of mutation | Nucleotide in *D. dendriticus* | Nucleotide in *D. ditremus* | |
|---|---|---|---|---|
| 1 | 20 | A/G | G | |
| 2 | 21 | C/T | T | |
| 3 | 48 | T | C/T | |
| 4 | 52 | **T** | **C** | |
| 5 | 56 | G | A/G | |
| 6 | 60 | C/T | C | |
| 7 | 72 | C/T | C | |
| 8 | 75 | **G** | **T** | |
| 9 | 102 | C/T | C | |
| 10 | 109 | **A** | **G** | |
| 11 | 129 | C/T | C/T | |
| 12 | 135 | **G** | **A** | **FORWARD PRIMER** |
| 13 | 141 | **A** | **G** | *D. dendriticus*-specific: Dde_cob_F1 |
| 14 | 144 | **T** | **A** | *D. ditremus*-specific: Ddi_cob_F1 |
| 15 | 156 | **G** | **A** | |
| 16 | 171 | **T** | **C** | |
| 17 | 177 | **G** | **A** | |
| 18 | 192 | A/G | A/G | |
| 19 | 201 | **T** | **C** | |
| 20 | 204 | A/G | A | |
| 21 | 207 | **T** | **C** | |
| 22 | 210 | T | C/T | |
| 23 | 225 | **C** | **T** | |
| 24 | 228 | **A** | **G** | |
| 25 | 247 | **A** | **G** | |
| 26 | 250 | C/T | C | |
| 27 | 253 | T | C/T | |
| 28 | 261 | **T** | **C** | |
| 29 | 276 | **A** | **G** | |
| 30 | 277 | C/T | T | |
| 31 | 279 | A/T | G | |
| 32 | 282 | C/T | T | |
| 33 | 285 | C/T | T | |
| 34 | 294 | **T** | **C** | |
| 35 | 309 | A/G | A/G | |
| 36 | 324 | **T** | **C** | |
| 37 | 348 | **G** | **T** | |
| 38 | 363 | A/G/T | C | |
| 39 | 369 | **A** | **C** | |
| 40 | 372 | T | C/G | |
| 41 | 378 | A/G | A | |
| 42 | 384 | A/G | A | |
| 43 | 393 | C/T | T | |
| 44 | 396 | **A** | **G** | |
| 45 | 408 | **T** | **G** | **REVERSE PRIMER** |
| 46 | 417 | **T** | **A** | *D. dendriticus*-specific: Dde_cob_R1 |
| 47 | 423 | **T** | **A** | *D. ditremus*-specific: Ddi_cob_R1 |
| 48 | 430 | **A** | **G** | |
| 49 | 432 | A | A/G | |
| 50 | 435 | T | C/T | |
| 51 | 444 | T | A/G | |
| 52 | 447 | **C** | **T** | |
| 53 | 450 | T | C/T | |
| 54 | 463 | T | C/T | |
| 55 | 468 | G | A/G | |
| 56 | 471 | **G** | **T** | |
| 57 | 474 | G/T | T | |
| 58 | 486 | **T** | **C** | |
| 59 | 495 | T | C/T | |
| 60 | 501 | C/T | T | |
| 61 | 504 | **A** | **G** | |
| 62 | 507 | **T** | **G** | |
| 63 | 510 | **C** | **T** | |
| 64 | 522 | G | A/G | |
| 65 | 528 | **A** | **G** | |
| 66 | 531 | A/G | G | |

| | | | |
|---|---|---|---|
| 67 | 540 | C/T | T |
| 68 | 546 | C/T | T |
| 69 | 547 | T | C/T |
| 70 | 549 | **G** | **A** |
| 71 | 553 | A/G/T | T |
| 72 | 564 | A | A/G |
| 73 | 585 | T | A/G |
| 74 | 588 | A/G | T |
| 75 | 615 | C/T | T |
| 76 | 624 | C | C/T |
| 77 | 627 | **G** | **A** |
| 78 | 630 | **A** | **G** |
| 79 | 633 | C | C/T |
| 80 | 650 | C/T | C |
| 81 | 657 | A/G | G |
| 82 | 663 | T | C/T |
| 83 | 666 | C | C/T |
| 84 | 669 | C/T | T |
| 85 | 675 | G/T | G |
| 86 | 678 | A/T | T |
| 87 | 688 | T | C/T |
| 88 | 690 | T | G/A |
| 89 | 696 | **C** | **A** |
| 90 | 702 | A/T | T |
| 91 | 708 | A/G | A |
| 92 | 709 | **G** | **A** |
| 93 | 712 | A/G | G |
| 94 | 714 | T | A/C |
| 95 | 726 | **G** | **A** |
| 96 | 727 | **G** | **A** |
| 97 | 732 | C/T | C |
| 98 | 738 | **G** | **A** |
| 99 | 739 | A/G | G |
| 100 | 741 | **T** | **C** |
| 101 | 745 | C/T | T |
| 102 | 750 | G | A/G |
| 103 | 753 | **C** | **T** |
| 104 | 762 | **G** | **A** |
| 105 | 765 | C/T | T |
| 106 | 770 | G/C | C |
| 107 | 771 | C/T | A |
| 108 | 772 | A/G | G |
| 109 | 798 | C/T | T |
| 110 | 805 | A/T | T |
| 111 | 807 | C/T | T |
| 112 | 813 | **T** | **C** |
| 113 | 822 | **C** | **T** |
| 114 | 825 | **T** | **A** |
| 115 | 831 | **G** | **A** |
| 116 | 834 | A/C/G/T | A |
| 117 | 837 | A/C/G | G |
| 118 | 846 | C | C/T |
| 119 | 853 | C/T | T |
| 120 | 859 | A/G | G |
| 121 | 882 | A/G | G |
| 122 | 885 | T | C/T |
| 123 | 888 | T | A/C |
| 124 | 891 | **T** | **C** |
| 125 | 900 | **A** | **T** |
| 126 | 912 | T | C/T |
| 127 | 933 | **A** | **T** |
| 128 | 936 | T | C/T |
| 129 | 940 | A/G | G |
| 130 | 942 | **G** | **T** |
| 131 | 945 | A | A/G |
| 132 | 951 | **C** | **T** |
| 133 | 952 | **T** | **C** |
| 134 | 954 | A | A/G |
| 135 | 972 | **C** | **T** |
| 136 | 975 | **A** | **T** |
| 137 | 978 | **T** | **G** |

**FORWARD PRIMER**
*D. dendriticus*-specific: Dde_cob_F2
*D. ditremus*-specific: Ddi_cob_F2

| | | | | |
|---|---|---|---|---|
| 138 | 993 | **A** | **G** | |
| 139 | 996 | C/T | C | |
| 140 | 1004 | C/T | T | |
| 141 | 1005 | **A** | **G** | |
| 142 | 1008 | A | A/G | |
| 143 | 1014 | C/T | T | |
| 144 | 1020 | **A** | **T** | |
| 145 | 1026 | **A** | **T** | |
| 146 | 1027 | **G** | **A** | |
| 147 | 1029 | **G** | **T** | |
| 148 | 1032 | **A** | **G** | |
| 149 | 1033 | A/G | A | |
| 150 | 1044 | A | A/G | |
| 151 | 1045 | A/T | A | |
| 152 | 1056 | **C** | **T** | |
| 153 | 1071 | **A** | **T** | **REVERSE PRIMER** |
| 154 | 1073 | **G** | **A** | *D. dendriticus*-specific: Dde_cob_R2 |
| 155 | 1074 | **C** | **T** | *D. ditremus*-specific: Ddi_cob_R2 |
| 156 | 1076 | **C** | **T** | |
| 157 | 1079 | **T** | **C** | |
| 158 | 1101 | **T** | **A** | |
| 159 | 1102 | T | T/G | |
| 160 | 1107 | A | A/G | |
